# Genome-wide analysis of respiratory burst oxidase homolog (*Rboh*) genes in *Aquilaria* species and insight into ROS-mediated metabolites biosynthesis and resin deposition

**DOI:** 10.3389/fpls.2023.1326080

**Published:** 2024-02-09

**Authors:** Khaleda Begum, Ankur Das, Raja Ahmed, Suraiya Akhtar, Ram Kulkarni, Sofia Banu

**Affiliations:** ^1^ Department of Bioengineering and Technology, Gauhati University, Guwahati, Assam, India; ^2^ Symbiosis School of Biological Sciences, Symbiosis International (Deemed University), Pune, India

**Keywords:** *Aquilaria*, *Rboh* proteins, ROS generation, secondary metabolites, agarwood

## Abstract

Respiratory burst oxidase homolog (*Rboh*) generates reactive oxygen species (ROS) as a defense response during biotic and abiotic stress. In *Aquilaria* plants, wounding and fungal infection result in biosynthesis and deposition of secondary metabolites as defense responses, which later form constituents of fragrant resinous agarwood. During injury and fungal invasion, *Aquilaria* tree generates ROS species via the Rboh enzymes. Despite the implication of *Rboh* genes in agarwood formation, no comprehensive genomic-level study of the *Rboh* gene family in *Aquilaria* is present. A systematic illustration of their role during stress and involvement in initiating signal cascades for agarwood metabolite biosynthesis is missing. In this study, 14 *Rboh* genes were retrieved from genomes of two *Aquilaria* species, *A. agallocha* and *A. sinensis*, and were classified into five groups. The promoter regions of the genes had abundant of stress-responsive elements. Protein–protein network and *in silico* expression analysis suggested their functional association with MAPK proteins and transcription factors such as WRKY and MYC2. The study further explored the expression profiles of *Rboh* genes and found them to be differentially regulated in stress-induced callus and stem tissue, suggesting their involvement in ROS generation during stress in *Aquilaria*. Overall, the study provides in-depth insight into two *Rboh* genes, *AaRbohC* and *AaRbohA*, highlighting their role in defense against fungal and abiotic stress, and likely during initiation of agarwood formation through modulation of genes involved in secondary metabolites biosynthesis. The findings presented here offer valuable information about Rboh family members, which can be leveraged for further investigations into ROS-mediated regulation of agarwood formation in *Aquilaria* species.

## Graphical Abstract

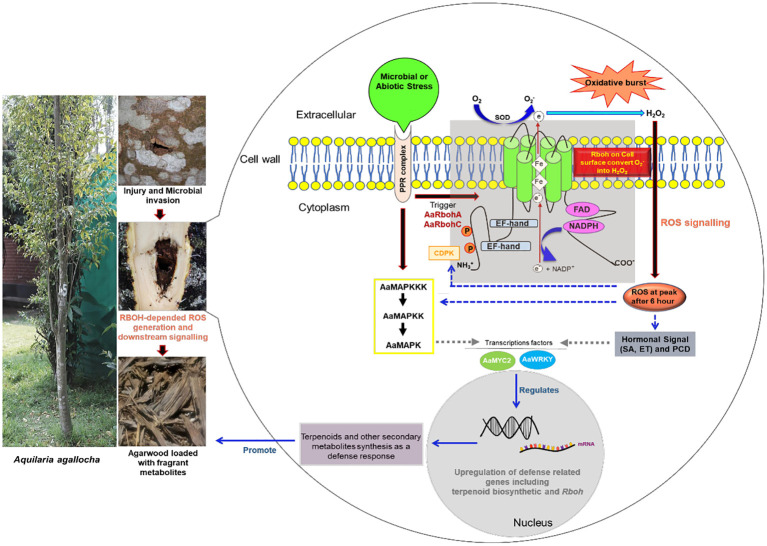

## 1 Introduction

The evolution of plants as sessile organisms presents a unique set of challenges. They are constantly exposed to various environmental stresses, both biotic (infection by bacteria, fungi, nematodes, etc.) and abiotic (drought, heavy metals, radiation, salinity, etc.), which can profoundly impact plant growth and yield (Wang et al., 2020; Mahalingam et al., 2021). Plant cells resist or respond to such stresses by promoting accumulation of reactive oxygen species (ROS). These species regulate almost all biological processes associated with developmental stages, stresses, and immunity responses (Castro et al., 2021). Although, ROS were once considered toxic by-products inside the cells because of cellular aerobic metabolism (Inupakutika et al., 2016). Recent studies affirm that they act as crucial signaling molecules to activate signal transduction cascades related to stress responses (Hu et al., 2018; Hawamda et al., 2020). However, beyond a specific threshold level, accumulation of ROS species can cause abnormal and irreparable metabolic changes and cell damage (Liu and He, 2016; Cheng et al., 2020; Wang et al., 2020). In plants, hydrogen peroxide (H_2_O_2_) is primary ROS species produced during C2 cycle in peroxisome (photorespiration) (Liu and He, 2016). In addition, as a by-product of photosynthesis and respiration, the photosynthesizing chloroplast and respiring mitochondria produce superoxide and hydrogen peroxide (Navathe et al., 2019). Superoxide anion (O^−^_2_) first generated from apoplastic molecular oxygen (O_2_) by the enzyme respiratory burst oxidase homolog protein (*Rboh*), which is next conFIGverted to H_2_O_2_ through superoxide dismutation reaction by the enzyme superoxide dismutase (Navathe et al., 2019). The different *Rboh* isoforms, also known as Nicotinamide Adenine Dinucleotide Phosphate Hydrogen (NADPH) oxidase in plasma membrane, transfer electrons from cytosolic NADPH/Nicotinamide Adenine Dinucleotide (NAD) + Hydrogen (H) (NADH) to apoplastic oxygen, producing the ROS in the cells (Yu et al., 2020). Plant Rboh is an intrinsic protein with six conserved transmembrane helices containing two basic helix-loop-helix calcium-binding structural domains (EF-hands) that are directly controlled by Ca^2+^ ions (Yu et al., 2020). Plant Rboh proteins share structural and functional domains with the mammalian homolog catalytic unit gp91phox, with the exception of an extended N-terminal sequence (Cheng et al., 2013; Cheng et al., 2019). The extended N-terminal region of plant Rboh contains two potential calcium-binding sites regulated by Ca^2+^ ion. The hydrophilic C-terminal domain has cytosolic-facing flavin adenine dinucleotide (FAD) and NADPH-binding sites. At the apoplast, heme groups are necessary for electron transport across the membrane to oxygen (O_2_, the electron acceptor) through FAD (Mahalingam et al., 2021).

Rboh of plants are a small multigene protein family (Marino et al., 2012). To date, genes encoding the Rboh proteins have been investigated and delineated in several plant species, namely, *Citrus sinensis* (Zhang et al., 2022); *Capsicum annuum* (Zhang et al., 2021); *Hordeum vulgare* (Lightfoot et al., 2008; Mahalingam et al., 2021); *Nicotiana tobacum* (Yu et al., 2020); *Prunus avium*, *Prunus dulcis*, *Malus domestica Rubus occidentalis*, *Fragaria vesca*, and *Rosa chinensis* (Cheng et al., 2020); *Tritium aestivum* (Hu et al., 2018; Navathe et al., 2019); *Glycine* max (Liu et al., 2019); *Oryza sativa* (Wong et al., 2007; Yamauchi et al., 2017); *Malus domestica* (Cepauskas et al., 2015); *Vitis vinifera* (Cheng et al., 2013); *Medicago truncatula* (Marino et al., 2011); *Zea mays* (Lin et al., 2009); *Arabidopsis thaliana* (Torres and Dangl, 2005; Torres et al.,1998); and *Lycopersicon esculentum* (Sagi and Fluhr, 2001). The rice OsRbohA was the first Rboh protein identified in plants (Navathe et al., 2019). The model plant *Arabidopsis thaliana* genome has 10 numbers of *AtRboh* genes, and, as per GeneVestigator microarray datasets (Zimmermann et al., 2004), of these, *AtRbohH* and *AtRbohJ* are involved with the growth of tip of pollen tube, whereas *AtRbohA*, *AtRbohB*, *AtRbohC*, *AtRbohG*, *AtRbohE*, and *AtRbohI* are expressed in root tissues; *AtRbohD* and *AtRbohF* are expressed across all *A*. *thaliana* tissues (Hawamda et al., 2020). On the other hand, *AtRbohB* was responsible for seed ripening and root hair formation, whereas *AtRbohC* controlled growth of the root hair cell (Navathe et al., 2019). Expression of *AtRbohD* and *AtRbohE* was also induced by plant hormone jasmonic acid, indicating their role in stress response and signaling (Maruta et al., 2011). AtRbohE was also reported to regulate the tapetal programmed cell death (PCD) and pollen formation in wheat (Hu et al., 2018).

In *Aquilaria* plants, as a result of biotic and abiotic stress, heartwood of the tree transforms into worthy resinous dark wood known as agarwood (Das et al., 2021). In general, *Aquilaria* species are diploid in nature. The genome size of *A*. *sinensis* is 726.5 Mb (Ding et al., 2020) and of *A*. *agallocha* is 736 Mb (Chen et al., 2014). Agarwood is well-known around the world for its usage as a primary ingredient in perfume, incense, and medicine (Monggoot et al., 2017). It has been traded and utilized for centuries to create perfume, which is still employed in religious and cultural ceremonies (López-Sampson and Page, 2018; Barden et al., 2000). Global agarwood prices can range from US$ 20 to US$ 6,000/kg for wood chips, depending on quality, or US$ 10,000/kg for the actual wood (Abdin, 2014). Agarwood essential oil can also fetch up to US$ 30,000/kg. According to estimates, the agarwood market in the world is worth between $6 and $8 billion annually (Tan et al., 2019). Among all the species of this genus, *A. agallocha* and *A. sinensis*, are known for producing high quality agarwood (Kristanti et al., 2018). When the tree is physiologically triggered by physical wound, followed by insect invasion or microbial infection, it activates defense-related signal transduction pathways, leading to accumulation of fragrant metabolites (Liu et al., 2013; Mohamed et al., 2014; Tan et al., 2019). Sesquiterpenes and 2-(2-phenylethyl)chromones are the two prominent chemical types found to be deposited in agarwood (Naef, 2011; Yang et al., 2021; Zhang et al., 2013). In addition, H_2_O_2_ burst (ROS production) is known to occur in wounded *Aquilaria* trees, leading to deposition of resins loaded with these metabolites (Zhang et al., 2013). Also, in plants, H_2_O_2_ is known to play a role in the regulation of the biogenesis of the secondary metabolites, *viz*., capsodiol, phenolics, and β-coumaroyl octopamine in tobacco, carrot, and potato, respectively (Matsuda et al., 2001). Previous studies have established role of *Rboh* gene families in ROS molecules accumulations after microbial invasion in plants (Morales et al., 2016; Chang et al., 2020; Pacheco-Trejo et al., 2022). Because agarwood resin formation in *Aquilaria* tree is an outcome of microbe-mediated stress, it leads us to hypothesize that, during microbial infections, *Aquilaria* trees accumulate ROS through the action of Rboh proteins. The ROS produced initiate a cascade of biochemical reactions that activate the defense-related signal transduction processes, eventually activating secondary metabolite biosynthetic genes for defense responses (Xu et al., 2016; Tan et al., 2019; Das et al., 2021).

To the best of our knowledge, a comprehensive genome-level illustration of the *Rboh* family members and their role during stress, and relation with downstream cascades leading to secondary metabolite biosynthesis is missing in *A. agallocha*. Therefore, this study aims to systemically identify, characterize, and analyze the expression of the *Rboh* genes in stress-induced tissues, which will likely identify the key members involved in ROS generation. In addition, findings in this current study will lay the foundation for understanding the molecular basis and regulatory mechanisms of *Aquilaria* species *Rboh* genes and their possible involvement in secondary metabolite biosynthesis and agarwood resin deposition.

## 2 Materials and methods

### 2.1 Sequence retrieval and identification of *Rboh* genes

The genomic sequence data of *A. agallocha* and *A. sinensis* were collected from previous annotation projects (Das et al., 2021 and Ding et al., 2020). Following that the sequence alignment of respiratory burst NADPH oxidase (PF08414), ferric reductase-like transmembrane component (PF01794), FAD-binding (PF08022), and ferric reductase NAD (PF08030) were obtained from the Pfam database and were used to build hidden Markov model (HMM) profile utilizing hmmbuild in the software HMMER 3.3.2 (Potter et al., 2018). Subsequently, the hmmsearch program was utilized to identify the putative *AaRboh*s and *AsRboh*s proteins, and the redundant protein sequences were discarded. All the putative sequences were further confirmed through Pfam database (http://pfam.xfam.org/) and SMART database (http://smart.embl-heidelberg.de/) for the presence of conserved NADPH_Ox (PF08414) domain (Cheng et al., 2020; Zhang et al., 2023). The physiological properties such as molecular weight (kDa), isoelectric point (pI), instability index (II), aliphatic index Ai), and the grand average of hydropathicity (GRAVY) were calculated by using the ExPASy-ProtParam tool (http://web.expasy.org/protparam/). The subcellular location of the Rboh proteins was predicted using online web server CELLO version 2.5 (http://cello.life.nctu.edu.tw/).

### 2.2 Multiple sequence alignments and phylogenetic analysis

Sequences of Rboh proteins of *Solanum tuberosum*, *Arabidopsis thaliana*, *Hordeum vulgare*, *Oryza sativa*, and *Glycine max* were downloaded from UniProtKB and aligned with predicted AaRboh and AsRboh protein sequences in the MEGA-X program (https://www.megasoftware.net/). The sequence alignment was presented with ESPrit 2.2-ENDscript 1.0 (Robert and Guoet, 2014). A phylogenetic tree was constructed on the basis of the alignment in the MEGA-X program using neighbour-joining method with parameter set as P distance model and 1,000 bootstrap replicates (Kumar et al., 2016).

### 2.3 Gene structure, *cis*-acting elements analysis

The intron–exon structure of individual *Rboh* genes was predicted utilizing genomic DNA and complete coding sequence (CDS) in Gene structure Display Server GSDS v2to.0 (http://gsds.cbi.pku.edu.cn) (Hu et al., 2015). The *cis*-acting regulatory elements were identified in 2.4 kb upstream of each gene using PlantCARE database.

### 2.4 Conserve motif in the proteins and homology modeling

Conserved motifs in the *Rboh* genes were predicted utilizing the MEME suite (http://meme-suite.org/). The analysis parameters were configured to identify the top 10 conserved motifs, whereas the remaining settings were kept at their default (Bailey et al., 2009) (http://bioinformatics.psb.ugent.be/webtools/plantcare/html/) (Lescot et al., 2002). Homology modeling was employed to determine the 3D structures of the Rboh proteins using Swiss Model web server (https://swissmodel.expasy.org/). Structure assessment of the modeled structures was done considering the Ramachandran Plot and Stereochemistry (MolProbity score) and Clash score parameters (https://swissmodel.expasy.org/assess). Geometric and energetic validation of the structures was done using ERRAT server of SAVES v6.0 (https://saves.mbi.ucla.edu/).

### 2.5 Synteny and duplication analyses

To identify synteny blocks within the two *Aquilaria* genomes and with other plants (*A. thaliana* and *S. tuberosum*), blastp and Quick MCScanX Wrapper were employed and later visualized with the Dual Synteny plotter in TBtools (Chen et al., 2020). Duplicated genes were identified using *DupGen-finder* software (https://github.com/qiao-xin/DupGen_finder) using *A. thaliana* as outgroup and subsequently classified into duplication type following default parameters as per the user manual (Qiao et al., 2019). The non-synonymous substitution rate (*Ka*), synonymous substitution rate (*Ks*), and *Ka/Ks* ratio were calculated with TBtools (Wang et al., 2010). Divergence duration for duplication of paralogs pairs of the gene was calculated as per the formula T = Ks/2λ (where λ indicates the clock-of-like rate of 6.96 synonymous substitutions per 10^−9^ years) (Lopez-Ortiz et al., 2019).

### 2.6 Functional predictions and protein–protein interactions

Probable functions of Rboh proteins were predicted on the basis of assignment of Gene Ontology (GO) terms and Kyoto Encyclopedia of Genes and Genomes (KEGG) annotations with e-value cutoff < 10^−5^. Regulatory network and their functional partners were identified through STRING v11.5 program with the following terms: databases, experimental evidences, gene neighborhood, gene co-occurrence gene fusion, co-expression, textminig, co-expression, and protein homology parameters utilizing *Arabidopsis* homologous proteins as reference.

### 2.7 The expression patterns of the *AaRboh* and *AsRboh* genes

To study transcript abundance of *Rboh* genes, RNA-seq data were downloaded from NCBI-SRA website (https://www.ncbi.nlm.nih.gov/sra). The transcript abundance of *AaRboh* genes in agarwood (SRX4149019-SRX4149021) and healthy (SRX4184708-SRX4184710) wood tissues were calculated and compared. Similarly, transcript abundance of *AsRboh* genes in the different tissues/organs, *viz*., aril (SRX6871071 and SRX6871068), seed (SRX6871057 and SRX6871070), flower (SRX6871060 and SRX6871063), bud (SRX6871059 and SRX6871062), leaf (SRX6871066 and SRX6871058), salinity stressed callus (SRX1495981 and SRX1495736), flower (SRX6871059 and SRX6871062), and wounded stem (SRX6871056 and SRX6871064), was accessed. First, the short reads were aligned to the genome using HISAT2 (Kim et al., 2015), following the reads were assembled and quantified using StringTie (Kovaka et al., 2019). Differentially expressed genes were then identified using DESeq2 software (Love et al., 2014).

### 2.8 Plant material, growth, and treatment

*A. agallocha* calli were induced from leaves on Murashige–Skoog (MS) medium supplemented with dichlorophenoxyacetic acid (6 mg/L) and kinetin (2 mg/L). The calli were transferred to fresh MS medium every month until the formation of friable calli. To induce stress, the calli were put into an MS medium containing 10 mM H_2_O_2_ and exposed to 5 mM dimethylthiourea (DMTU; an H_2_O_2_ scavenger) and combination of H_2_O_2_ with DMTU separately (Wang et al., 2018). Calli, without any treatment, were considered as control. After treatment, the samples were harvested at 0 h, 1 h, 2 h, 6 h, 12 h, 24 h, and 48 h.

Healthy saplings of *A. agallocha* maintained in pots at the Bioengineering and Technology Department of Gauhati University were selected for stress treatments as per standard methodology (Lv et al., 2019). The lateral stems were cut with scissors, and 1 cm from the apical end of the cut lateral stems was immersed separately in distilled H_2_O, H_2_O_2_, and DMTU solutions for stress treatments. The healthy *Aquilaria* lateral stems were taken as a control. After that, the portions immersed in treatment solutions were discarded, and the remaining treated stems (approximately 2 cm) were exposed to air for sample harvesting. Samples were harvested after 0 h, 1 h, 2 h, 6 h, 12 h, 24 h, and 48 h of air exposure. Treatment of seedlings after cutting refers to physical wounding. Wood samples (resin-embedded infected wood and healthy wood of *A*. *agallocha*) from Hoollongapar Gibbon Sanctuary in Jorhat, Assam, India, were collected following the methodology described by Islam et al. (2020) to analyze the *AaRboh* transcripts abundance. All sample sets were rapidly immersed in the liquid nitrogen and stored at −80°C until experiments were done.

### 2.9 RNA extraction and real-time reverse transcription PCR analysis

Total RNA from stem tissues was extracted following the RNA extraction method outlined by Islam and Banu (2019) and from callus tissues using the RNeasy Plant Mini Kit (Qiagen). The quality and concentration of extracted RNA were assessed with 1% agarose gel electrophoresis and estimated with the Multiskan Sky Microplate Spectrophotometer (Thermo Fisher Scientific, USA), respectively. One micrograms of RNA was used to synthesize the first strand of cDNA with SuperScript III Reverse Transcriptase (Thermofisher). The qRT-PCR was carried out using a QuantStudio™ 3 real-time PCR system (Applied Biosystems, USA) and the PowerUp SYBR Green Master Mix (Applied Biosystems). Seven *AaRboh*s gene-specific primer pairs were designed with PrimerQuest software of IDT (https://sg.idtdna.com/pages/tools/primerquest/) and are enlisted at **Supplementary Table 1**. The standardized *GAPDH* primer was utilized as the internal control (Islam et al., 2020). For each biological replicate, the analyses were performed with three technical replicates, each containing 20 µl of reaction volume in optical stripes, the temperature pattern of 95°C for 1 min, followed by 40 cycles at 95°C for 10 s and 60°C for 30 s, was followed as thermal cycler profile. Fold change in the gene expression was measured by the 2^ΔΔCt^ method (Ding et al., 2021).

### 2.10 ROS determination of treated plant materials

The endogenous ROS production was determined according to Wang et al. (2018) with minor modifications. Plant samples (3 g of fresh weight) were subjected to individual and combined treatments with H_2_O_2_ and DMTU. The treated samples were homogenized in 3 mL of pre-cooled acetone using a mortar and pestle on ice. Obtained mixtures were centrifuged at 3,000 rpm for 10 min at 4°C. The supernatant obtained (0.1 mL) was quickly mixed with 0.1 mL of 5% TiSO_4_ and 0.2 mL of NH_4_OH was added to it. The resulting mixtures were centrifuged at 3,000 rpm for 10 min at 4°C to separate the titanium–hydroperoxide complex precipitate, and the supernatants were discarded. After three washes with pre-cooled acetone, the precipitates were dissolved in 2 mL of H_2_SO_4_ (2 mol/L). Absorbance of solution at 415 nm was monitored to quantify H_2_O_2_ content. Obtained absorbances were compared with the calibration curve derived from known concentrations of H_2_O_2_ (30%) (Wang et al., 2018; Zhang et al., 2021).

### 2.11 Statistical analysis

Three each biological and technical replicate were used for each control and treatment samples. T-test was performed to validate the expression differences of the *Rboh* genes in the different treated conditions. The P-value cutoff ≤ 0.05 was considered to be statistically significant test result. The methodology followed in the current study was respresented as **Supplementary Figure 1**.

## 3 Results

### 3.1 Identification of *Rboh* Genes in *A*. *agallocha* and *A. sinensis*

Seven genes each from *A. agallocha* (*AaRboh*) and *A*. *sinensis* (*AsRboh*) were identified and characterized (**Supplementary Figure 2**). The *AsRboh* genes were distributed on to four chromosomes (Chr02, Chr04, Chr06, and Chr07) and *AaRbohs* on seven scaffolds (KK901300.1, KK899295.1, KK902390.1, KK900302.1, KK899913.1, KK900079.1, and KK899008.1). The length of the protein varied, i.e., the shortest and the longest Rboh belonged to *A. agallocha*. For example, AaRbohJ had 663 amino acids (shortest), and AaRbohA had 946 amino acids (longest) (**Table 1**). Molecular weights varied from 75.72 kDa (AaRbohJ) to 107.77 kDa *(*AsRbohA) and pI from 8.93 (AsRbohC2) to 9.45 (AaRbohA). GRAVY values, representing the grand average of hydropathicity of the Rboh proteins were found to be < 0, which indicated their hydrophilic nature.

**Table 1:** Details of A. agallocha Rboh (AaRboh) and A. sinensis Rboh (AsRboh) identified in the genomes and properties of their deduced proteins.

Gene name	Chromosome/ scaffold position	Start position	End position	Protein length (aa)	Molecular weight (kDa)	pI	Instability index	Aliphatic index	Asp + Glu	Arg + Lys	Grand average of hydropathicity (GRAVY)
***AsRbohC1***	Chr07	52648308	52656837	918	102.97	9.02	37.19	65.36	101	115	−0.253
***AsRbohC2***	ContigUN	1179685	1188148	884	99.34	8.93	37.72	66.51	97	111	−0.247
***AaRbohC***	KK899295.1	190500	198939	675	76.57	9.3	36.8	64.3	66	87	−0.139
***AsRbohD***	Chr07	35508180	35511918	874	99.09	9.15	41.29	73.43	92	110	−0.222
***AaRbohD***	KK902390.1	39399	43132	874	98.98	9.15	40.36	71.57	91	109	−0.21
***AsRbohB***	Chr06	2272107	2276522	887	101.08	9.34	42.8	76.26	91	115	−0.279
***AaRbohB***	KK900302.1	52418	56833	764	87.41	9.4	44.85	80.3	80	103	−0.283
***AsRbohA***	Chr02	62487359	62495094	946	107.77	9.3	51.06	92.82	99	122	−0.249
***AaRbohA***	KK899913.1	205958	213711	854	97.89	9.45	50.61	91.77	85	111	−0.265
***AsRbohE***	Chr04	77333078	77338035	926	104.915	9.07	47.25	85.43	98	113	−0.217
***AaRbohE***	KK899008.1	71417	76374	826	93.42	9.42	48.15	86.88	83	106	−0.18
***AaRbohF***	KK900079.1	48552	57956	777	88.94	9.2	49.85	90.5	72	90	−0.19
***AsRbohH***	Chr04	16169597	16174544	880	100.53	9.35	43.44	77.53	82	111	−0.183
***AaRbohJ***	KK901300.1	65448	70313[	663	75.72	9.22	45.33	81.44	69	86	−0.25

Rboh, respiratory burst oxidase homolog; AaRboh, Aquilaria agallocha respiratory burst oxidase homolog; ROS, reactive oxygen species; DMTU, dimethylthiourea; HMM, hidden Markov model; CDS, coding sequence; GO, Gene Ontology; KEGG, Kyoto Encyclopedia of Genes and Genomes database.

### 3.2 Multiple sequence alignment and phylogenetic analysis

**Figure 1:**
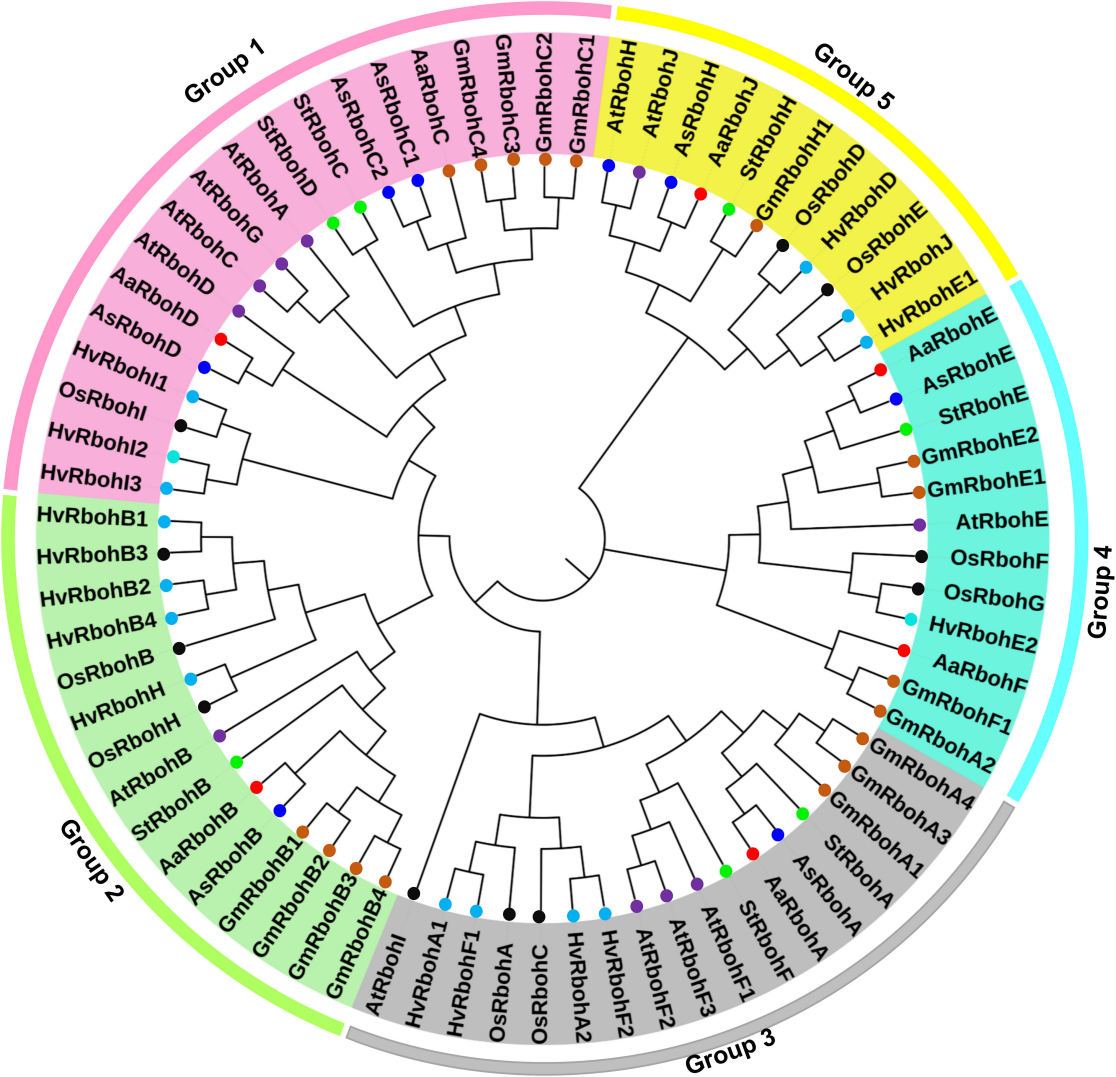
Phylogenetic relationship between the Rboh proteins of (*A*) *agallocha*, *(A) sinensis*, *(A) thaliana*, *(G) max*, *(H) vulgare*, and *S. tuberosum.* Molecular phylogenetic tree constructed using MEGA-X with NJ method–based P distance substitutions model. Bootstrap values used to assess the tree. Five group are shown as Groups 1–5 with different colors.

To determine the phylogenetic positions, a tree was build using 14 *Aquilaria* Rboh and 5, 12, 16, and 16 Rboh proteins of *S. tuberosum*, *A. thaliana*, *H. vulgare*, and *Glycine max*, respectively. Interestingly, RbohA-E were found in both the species, whereas RbohF and RbohJ were found in *A. agallocha* and RbohH in *A. sinensis*, respectively. In addition, *A. sinensis* had two members of RbohC, whereas *A. agallocha* had only one. The members of six plant species were majorly grouped into five major clades (**Figure 1**). The highest numbers of Rboh members were present in Group 1 (19), followed by Group 3 (17), Group 2 (15), Group 4 (12), and Group 5 (11). Group 1 composed of two AaRboh (AaRbohD and AaRbohC) and three AsRboh (AsRbohD, AsRbohC1, and AsRbohC2) proteins. Whereas, Group 2 composed of one each of AaRboh (AaRbohB) and AsRboh (AsRbohB). Similarly, Group 3 had one each of AaRboh (AaRbohA) and AsRboh (AsRbohA). Group 4 included two AaRboh proteins (AaRbohE and AaRbohF), one AsRboh (AsRbohE), one AaRboh (AaRbohJ), and one AsRboh (AaRbohH). All five groups contained at least one member from each of the six plant species, including *A. agallocha* and *A. sinensis*.

### 3.3 Subcellular location and gene structure

**Figure 2:**
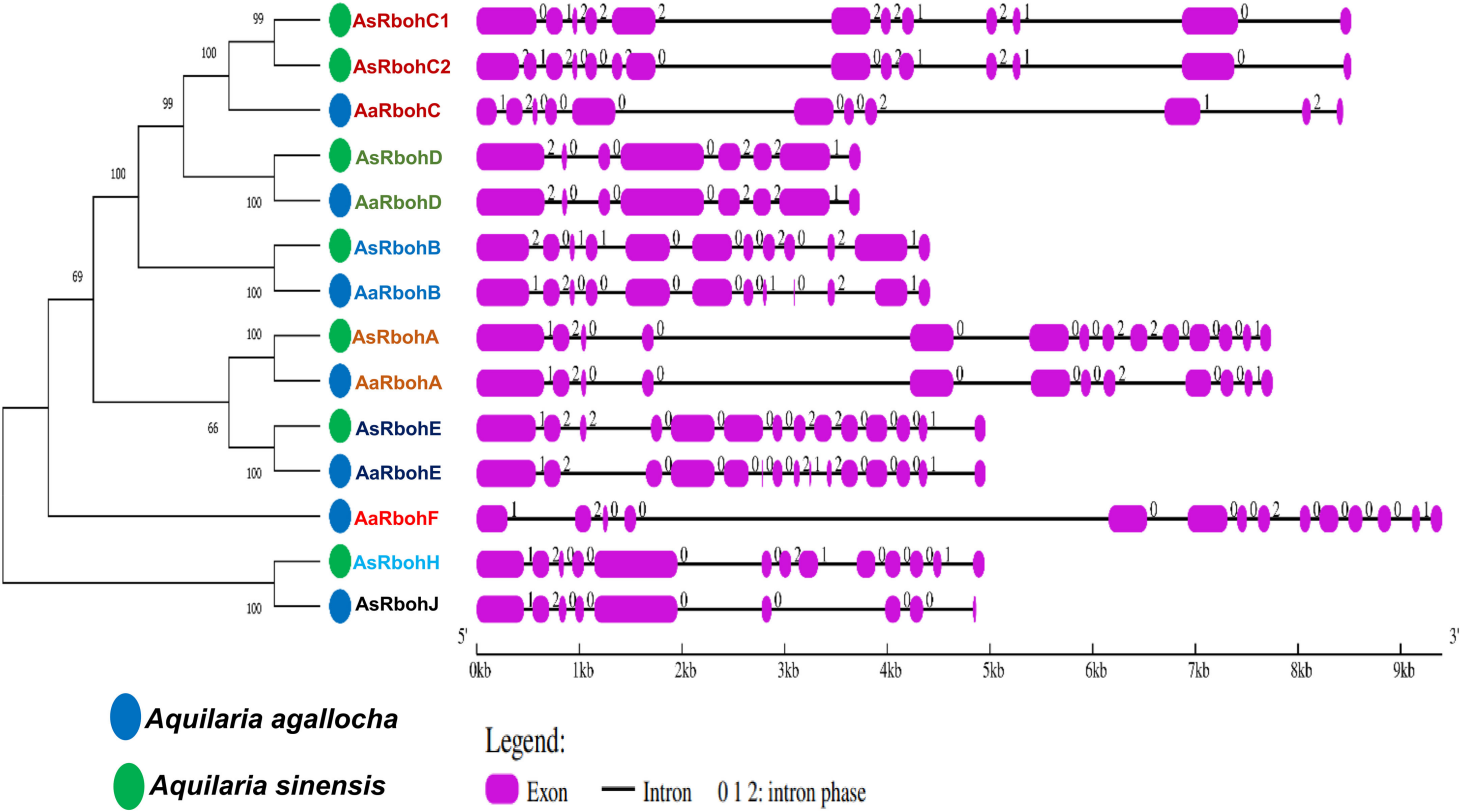
Schematic representation of structures of 14 putative *Rboh* genes in two *Aquilaria* species. The exons and introns are indicated with pink rectangles and black color lines, respectively, on the right of phylogenetic tree. The numbers (0, 1, and 2) on the gene structures indicate the intron phases.

The results of subcellular location prediction indicated that all Rboh proteins are localized to the plasma membrane. Furthermore, the structural organization of exon–intron sequences in the clustered genes displayed notable similarities, suggesting a close evolutionary relationship among them. They exhibited varying numbers of exons, ranging from eight (*AaRbohD* and *AsRbohD*) to 15 (*AaRbohE*). Most *Rboh* genes, on the other hand, contained either 12 (*AaRbohB*, *AaRbohA*, and *AsRbohC1*) or 14 (*AaRbohF*, *AsRbohB*, *AsRbohC2*, *AsRbohA*, and *AsRbohE*) exons (**Figure 2**). In terms of intron composition, the members showed variability in both the number and types of introns, where *AaRbohE* had the maximum intron. Phase 0 introns were the most abundant, totaling 81, followed by phase 2 introns being 42 and phase 1 introns being 31. The presence of phase 0 introns ranged from two to nine in each member, whereas phase 1 introns varied from one to three and phase 2 introns varied from two to three, with the exception of *AsRbohJ* (**Figure 2**).

### 3.4 *Cis*-acting elements of the putative *Rboh* promoter

**Figure 3:**
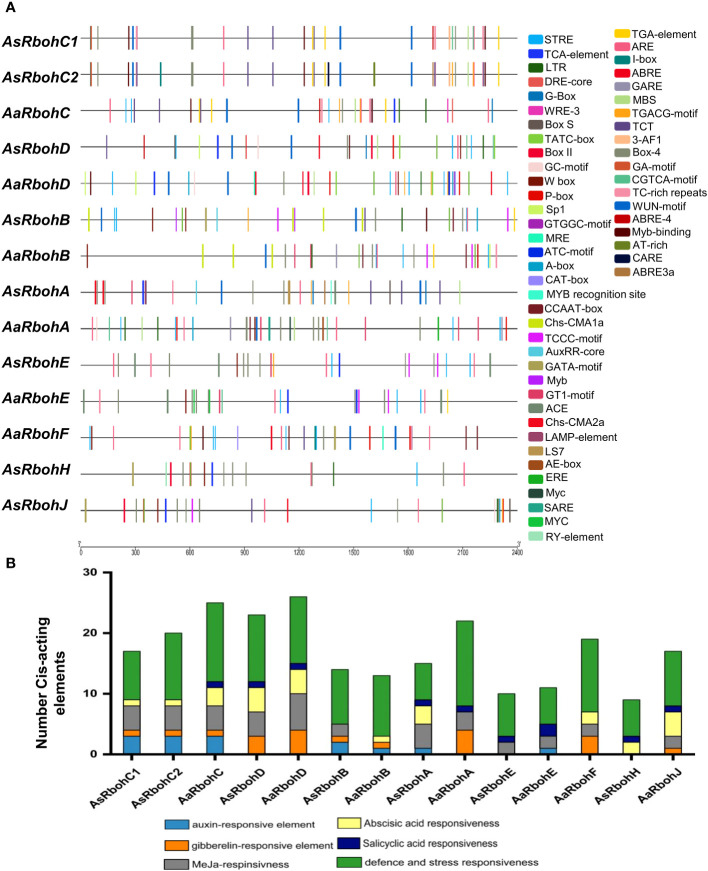
Distribution of major *cis*-acting elements in the promoter of *AaRboh* and *AsRboh* genes. **(A)** *Cis*-acting regulatory elements predicted in the 2.4-kb upstream regions of *AaRboh* and *AsRboh* genes, indicating with different color rectangular boxes. **(B)** The number of hormone responsiveness and defense-related *cis*-acting regulatory elements of *AaRboh* and *AsRboh* genes.

A total of 56 types of *cis*-acting elements were identified on the 2.4-kb region upstream of the translational start site of each *Rboh* genes (**Supplementary Table 2**). These elements were categorized into four major functional groups: hormone regulation, stress response, and metabolism-responsive and development-related *cis*-acting elements. Stress and defense responsiveness *cis*-acting elements were ARE (*cis*-acting elements for anaerobiosis), MBS (drought response), LTR (low-temperature responsive *cis*-acting element), TC-rich repeats (defense), and WUN motif (wound stress) (**Figure 3**). In addition, six types of plant hormone regulatory elements were salicylic acid response element (TCA-element and SARE); Gibberellin response element (TATC-box, P-box, and GARE); Auxin response element (AuxRR-core and TGA-element); Ethylene response element (ERE); Abscisic acid (ABA) response element (ABRE); and methyl jasmonate response elements (TGACG-motif and CGTAC-motif) (**Figure 3A**). The promoters also had cell differentiation and developmental processes elements such as RY-elements and CAT box *cis*-elements. Interestingly, the numbers of defense and stress responsiveness elements were observed in all *Rboh*s gene’s promoter in highest numbers, ranging from 6 (*AaRbohE*) to 14 (*AaRbohA*) (**Figure 3B**).

### 3.5 Gene location, synteny block analysis, and *Ka*/*Ks* calculation

**Figure 4:**
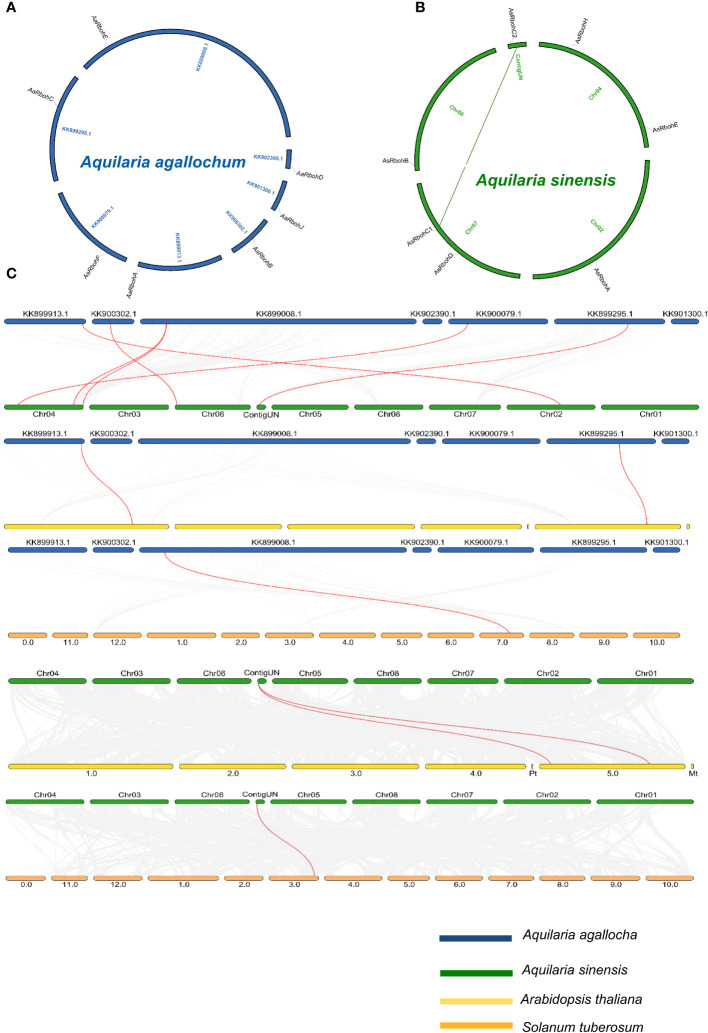
Overview of evolutionary relationship of *Rboh* of *A. agallocha*, *A. sinensis*, *A. thaliana*, and *S*. *tuberosum.* **(A)** Synteny analysis of *AaRboh*. **(B)** Synteny analysis of *AsRboh* and *AsRboh* (green line shows duplicate genes). **(C)** Synteny analysis among *A. agallocha* and *A. sinensis*; *A. agallocha* and *A. thaliana*; *A. agallocha* and *S. tuberosum*; *A. sinensis* and *A. thaliana*; and *A. sinensis and S. tuberosum.* Gray lines represent all collinearity blocks, whereas red lines show orthologous gene pairs among two species.

The seven *Rboh* genes of *A. agallocha* were distributed in seven different scaffolds (**Figure 4A**), and *AsRboh* genes were distributed in Chromosome 2 (*AsRbohA*), Chromosome 4 (*AsRbohE*), Chromosome 6 (*AsRbohB*), Chromosome 7 (*AsRbohC1* and *AsRbohD*), and ContigUN (*AsRbohC2*) (**Figure 4B**). The syntenic analysis unveiled a collinear relationship between five *AaRboh* genes, namely, *AaRbohA*, *AaRbohB*, *AaRbohC*, *AaRbohF*, and *AaRbohE* in *A. agallocha* and their counterparts in *A. sinensis* (**Figure 4C**). *AaRbohE* was found to be associated with two syntenic gene pairs in *A. sinensis*. The *AaRbohD* and *AaRbohJ* genes exhibited no collinear relationships with genes in *A. sinensis*. Furthermore, *AaRbohA* and *AaRbohC* displayed a collinear relationship with genes in *A. thaliana*, whereas *AaRbohE* exhibited synteny with *S*. *tuberosum*. The member *AsRbohC2* exhibited synteny with genes present in both *S. tuberosum* and *A. thaliana*. The syntenic blocks with their genome location were summarized in **Supplementary Table 3**. Duplication analysis indicated that, in *A. sinensis*, one gene pair *AsRbohC1* and *AsRbohC2* undergone segmental duplication, whereas *AsRbohD* emerged from the parental gene *AsRbohC1* through transposed duplication (TRD). Interestingly, in *A. agallocha*, three pairs of TRD genes were detected, where *AaRbohB*, *AaRbohC*, and *AaRbohE* duplicated from the parent gene *AaRbohA*. The Ka/Ks ratio of the duplicated gene pair was found to be < 1 (**Supplementary Table 4**). The divergent time of the duplicated members ranged from 1.4 to 227.85 million years ago (MYA).

### 3.6 Amino acid sequence and characteristic domain analysis of Rboh proteins

**Figure 5:**
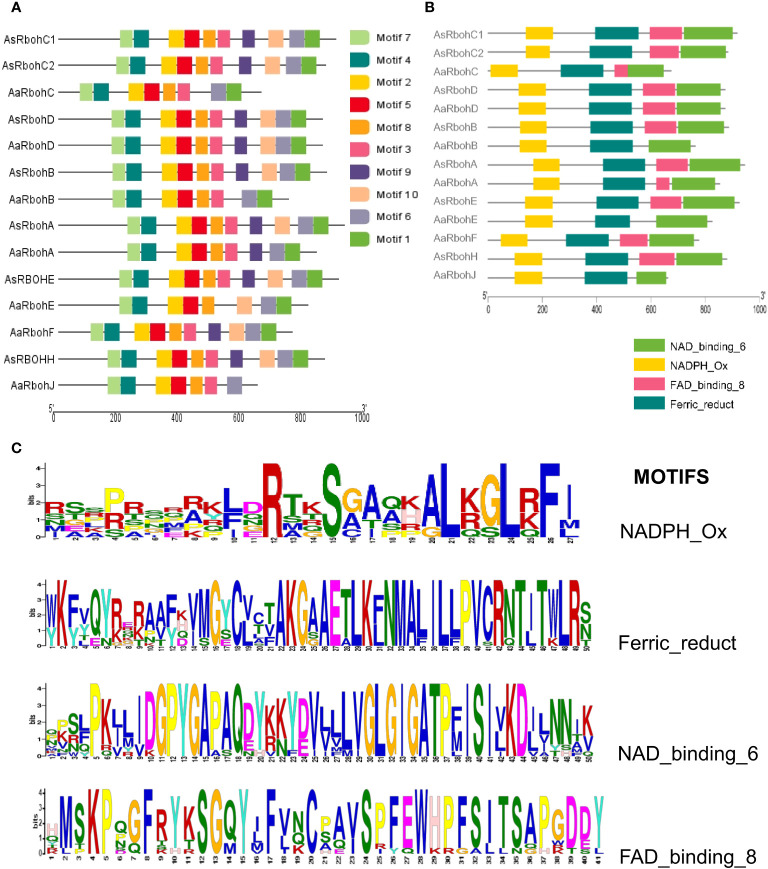
Conserved motifs distribution of AaRboh and AsRboh protein sequences. **(A)** Ten types of conserved motifs of AsRboh and AaRboh. **(B)** Four particular characteristics of motif of AsRboh and AaRboh. **(C)** Sequence logos of the NADPH_Ox, Ferric_reduct, FAD_binding_8, and NAD_binding_6 motif.

**Figure 6:**
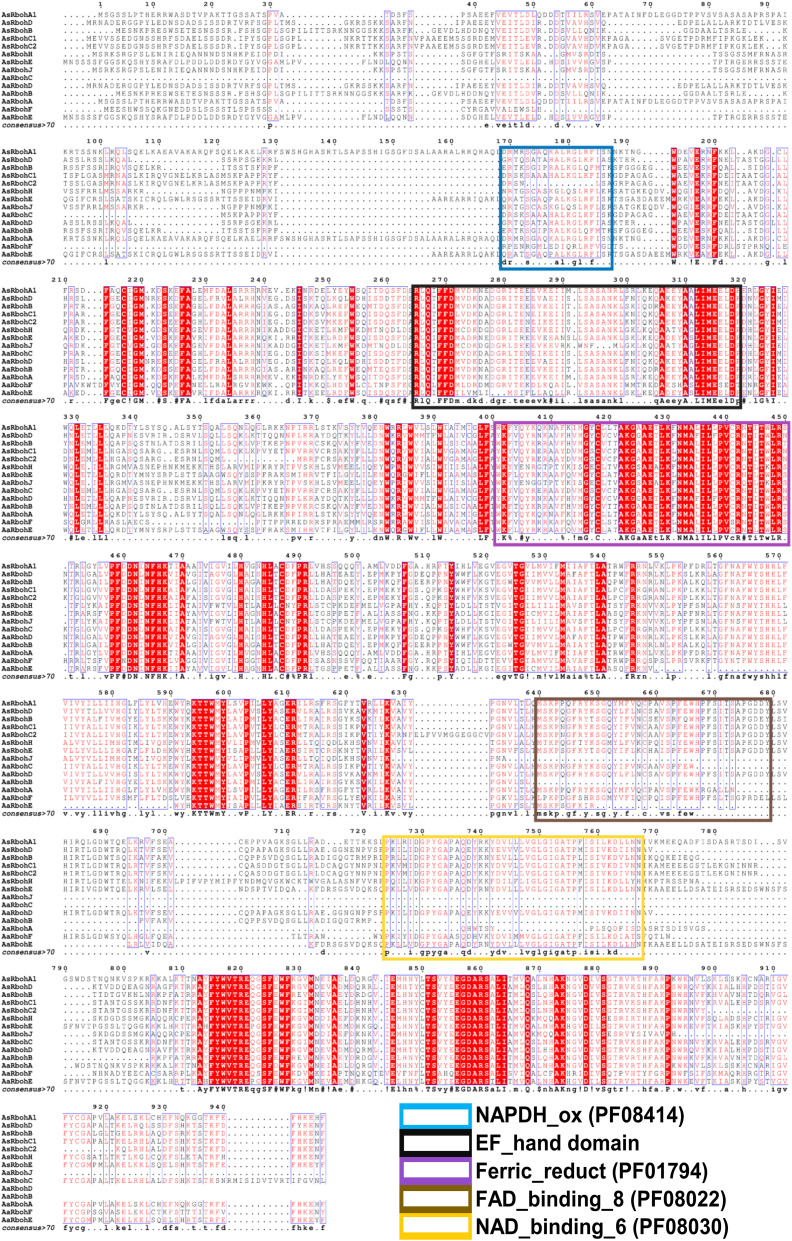
Multiple protein sequence alignment and domain structure of Rboh proteins of *A. agallocha* and *A. sinensis*. Highly conserved amino acids indicate with red shading, and low amino acid levels represent with lighter shading. The NADPH_ox (PF08414), EF-hand domain, Ferric_reduct (PF01794), FAD_binding_8(PF08022), and NAD_binding_6 (PF08030) were indicated with blue, black, violet, brown, and yellow color, respectively.

MEME suite tool identified 10 consensus motifs in the Rboh proteins based on degree of conservation amino acid residues. The motifs, *viz*., motif 1 (except AaRbohJ), motif 2, motif-3 (except AaRbohE), motif 4, motif 5, motif 6, motif 7, and motif 8, existed in all Rboh proteins (**Figure 5A**). Whereas, both motif 9 and motif 10 were missing in *Aquilaria* RbohB, RbohC, and RbohJ. The four conserved motifs typically found in Rboh proteins existed in the *Aquilaria* Rboh members (**Figure 5B**). The most conserved amino acid within these motifs were represented by higher bits size (**Figure 5C**). Multiple sequence alignment revealed the presence of characteristics conserved domains, i.e., NADPH oxidase (PF08414), EF hand, Ferric reductase (PF01794), FAD binding (PF08022), and NAD binding (PF08030) (**Figure 6**). However, NAD-binding domain was missing in AaRbohB, AaRbohF, and AaRbohJ, and Ca^2+^-binding EF-hand domain in AaRbohF. The Rboh protein’s motif analysis revealed that motif 7, motif 4, motif 2, motif 5, motif 8, and motif 3 are parts of the NADPH oxidase (PF08414); motif 8 and motif 3 are part of the transmembrane helix; motif 9 is a part of the FAD binding PF08022); and motif 10, motif 6, and motif 1 are parts of the NAD binding (PF08030) (**Supplementary Table 5**). Overall, few motifs in the Rboh members (except RbohA and RbohC) of *A agallocha* were missing.

### 3.7 Secondary and tertiary structures

The secondary structures (SSs) of the Rboh proteins consisted of α-helix, coils, turns, and β-sheet (**Supplementary Figure 3**). Among all, α-helices were seen to be most dominant. The conserved motifs were identified in the models, where NADPH_Ox, EF-hand, and Ferric-reduct appeared as α-helix. More than one type of SS was found in a few motifs. For example, NAD_binding_6 consisted of both α-helices and coils, and FAD_binding_8 composed of a β-strand and coils. Note that the results of assessment parameters of the tertiary structures suggested a good quality of the models. For instance, the Mol Probity and Clash scores ranged from 0.96 to 1.98 and from 0.39 to 4.69. In addition, all the structures were Ramachandran favored with % above 92. The quality factor of the models calculated using ERRAT ranged from 84.13 to 96.5, indicating an acceptable quality of the constructed models.

### 3.8 Functional analysis and protein–protein interaction

**Figure 7:**
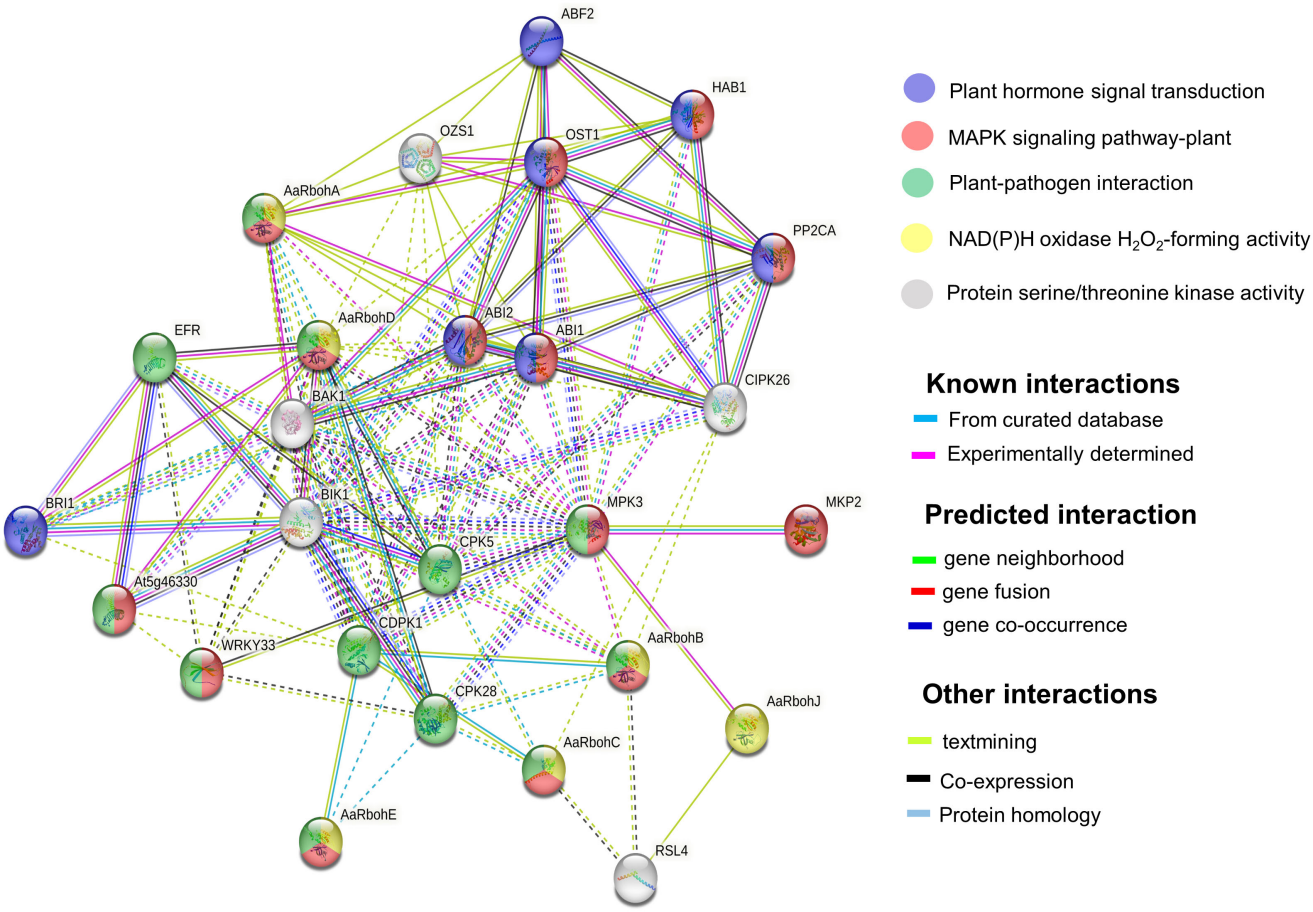
Protein interaction network of AaRboh in *A*. *agallocha* based on *Arabidopsis* orthologs. The potential AaRboh with their functional partners [MPK3 (mitogen-activated protein kinase 3), CPK28 (calcium-dependent protein kinase 28), CDPK1, WRKY33 (WRKY transcription factor 33), ERF (EF-TU receptor), BRI1 (brassinosteroid-insensitive 1), ABI1(abscisic acid–insensitive 1), ABI2, OST1(open stomata 1), ABF2 (abscisic acid–responsive element–binding factor 2), HAB (hypersensitive to ABA1), and PP2CA (protein phosphatase 2CA)] in each enriched pathway are displayed in a network model of proteins where the lines of various colors indicate the type of interactions between the potential AaRboh and their functional partners. The solid and dotted lines represent connections within the same and different clusters.

GO terms were assigned to the *Aquilaria* Rboh members, and their participation in biological processes (BP), molecular function (MF), and cellular component (CC) were elucidated (**Supplementary Table 6**). The string network model that consisted of 26 nodes and 121 edges (P = 1.0^e−16^) helped identify their functional partners (**Figure 7**). In addition, functional information pulled from KEGG database revealed their involvement in signal transduction pathways [mitogen-activated protein kinase (MAPK) signaling], plant–pathogen interaction, and plant hormone. In plant–pathogen interaction, AaRbohA, AaRbohB, AaRbohC, AaRbohD, and AaRbohE were directly involved and interacted with their functional partners, namely, MPK3, CPK28, CDPK1, WRKY33, and EFR. Similarly, the five Rboh proteins mentioned above were involved in MAPK signaling and interacted with their partners MKP2, MPK3, WRKY3, ABI1, ABI2, and OST1. AaRbohA and AaRbohD were possibly involved in hormone transduction and interacted with BRI1, ABI1, ABI2, OST1, ABF2, HAB1, and PP2CA (**Figure 7**).

### 3.9 *In silico* expression analysis of the *Rboh* genes and their functional partners in *A. agallocha* and *A. sinensis* tissues

**Figure 8:**
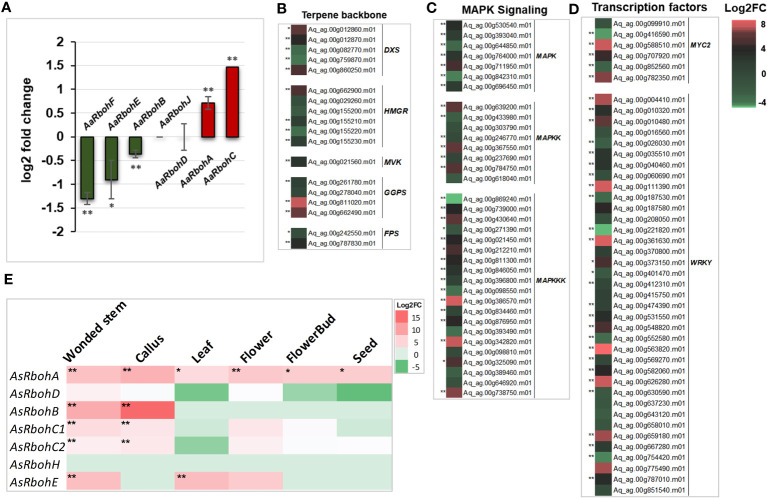
Expression profile of *AaRboh* and *AsRboh* genes of different types tissues of *A. agallocha* and *A. sinensis.* **(A)** *AaRboh* gene expression patterns in agarwood tissue. X-axis represents the *AaRboh* members, and Y-axis represents the log2 fold change value. **(B)** Expression patterns of genes involved in terpenoid biosynthesis genes where *DXS* indicates *1-deoxy-D-xylose-5-phosphate synthase*, *HMGR* indicates *3-Hydroxy-3-methylglutaryl-coenzyme A reductase*, *MVK* indicates *mevalonate kinase*, *GGPS* indicates *geranylgeranyl diphosphate synthase*, and *FPS* indicates *farnesyl pyrophosphate synthase.***(C)** Expression patterns of mitogen-activated protein kinase (*MAPK*) signaling cascades genes. **(D)** Expression pattern of transcription factors. **(E)** *AsRboh* gene expression patterns in the six different tissues compared to aril tissue. * indicates p-value less than 0.05, and ** indicates p-value less than 0.01.

Quantification of transcripts accumulation of the *Rboh* genes in *A. agallocha* showed differential upregulation of *AaRbohA* (0.7 log_2_FC) and *AaRbohC* (1.5 log_2_FC) in agarwood tissue. In contrast, *AaRbohB*, *AaRbohE*, and *AaRbohF* significantly downregulated by 1.3, 0.9, and 0.3 log_2_FC, respectively. At the same time, *AaRbohD* and *AaRbohJ* showed no change in expression (**Figure 8A**). The genes that act as transcription factors (*MYC2* and *WRKY*), in MAPK signaling cascade (*MAPK*, *MAPKK*, and *MAPKKK*) and in terpene backbone biosynthesis (*DXS*, *HMGR*, *MVK*, *GGPS*, and *FPS*), were significantly upregulated in agarwood tissue as shown in **Figures 8B–D**; **Supplementary Table 7**. In addition, expression of *AsRboh* in RNA-seq data of different tissues was estimated using aril tissue as control. Interestingly, *AsRbohA* was found to be significantly upregulated in all the tissues including wounded stem, callus, leaf, flower, and seed in the range of 4–8 log_2_FC (**Figure 8E**), whereas *AaRbohC1* and *AaRbohC2* upregulated only in wounded stem, callus, and flower in the range of 2–4 log_2_FC. Similarly, *AsRbohB* was comparatively higher in wounded stem (9 log_2_FC) and callus (15 log_2_FC), and *AsRbohE* in wounded stem (7.1 log_2_FC) and callus (7.3 log_2_FC). However, expression of *AsRbohD* and *AsRbohH* in the different tissues was either insignificant or had no difference (**Supplementary Table 8**).

### 3.10 Validation of the expression of *AaRboh* genes in H_2_O_2_-treated callus and stem

**Figure 9:**
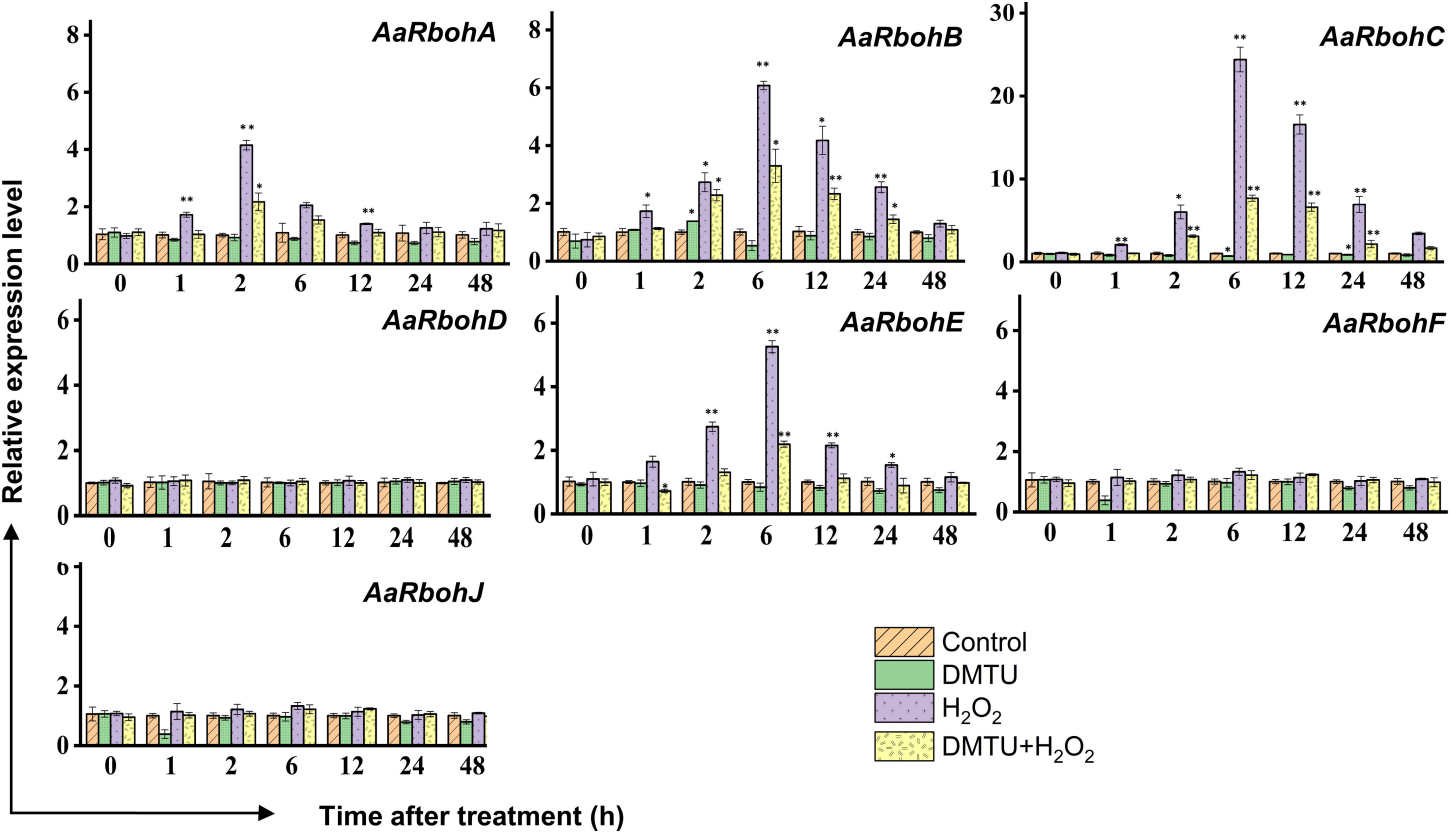
Relative expression levels of *AaRboh* genes in treated calli of *A*. *agallocha*. Relative transcripts abundance of seven *AaRboh* genes were measured in calli tissue transferred to MS media with H_2_O_2_, H_2_O_2_ + DMTU, DMTU, respectively, and calli without any treatment considered as control condition and samples harvested at 0 h, 1 h, 2 h, 6 h, 12 h, 24 h, and 48 h. Transcript abundances were measured using *A. agallocha GAPDH* as internal control. Asterisk (*) denotes a significant difference compared with healthy samples at 0.05 or **P < 0.01 (Student’s t-test). Data represent means ± SE off three independent experiments.

To evaluate the impact of hydrogen peroxide (H_2_O_2_) on the transcript levels of *AaRboh* genes, calli tissues were subjected to treatments involving H_2_O_2_, DMTU (a ROS scavenger), and combination of them (H_2_O_2_ + DMTU). In calli, the exposure to H_2_O_2_ resulted in the upregulation of *AaRboh* genes. Specifically, the expression of *AaRbohA* peaked at 2 h, showing a 4.15-fold increase. Similarly, the expression of *AaRbohB*, *AaRbohC*, and *AaRbohE* reached their peaks at 6 h, exhibiting 6.07-fold, 24.40-fold, and 5.26-fold increases, respectively. It is worth noting that there was a subsequent decline in the expression of these genes from 6 h to 48 h (**Figure 9**). When subjected to a combination of H_2_O_2_ and DMTU, the expression of these genes also increased, although not to the same extent as when induced by H_2_O_2_ alone. In contrast, treatment with DMTU alone resulted in lower expression compared to the control. Interestingly, there were no significant variations in the expression levels of the three genes, *AaRbohD*, *AaRBohF*, and *AaRbohJ*, when compared to the control across the different time periods.

**Figure 10:**
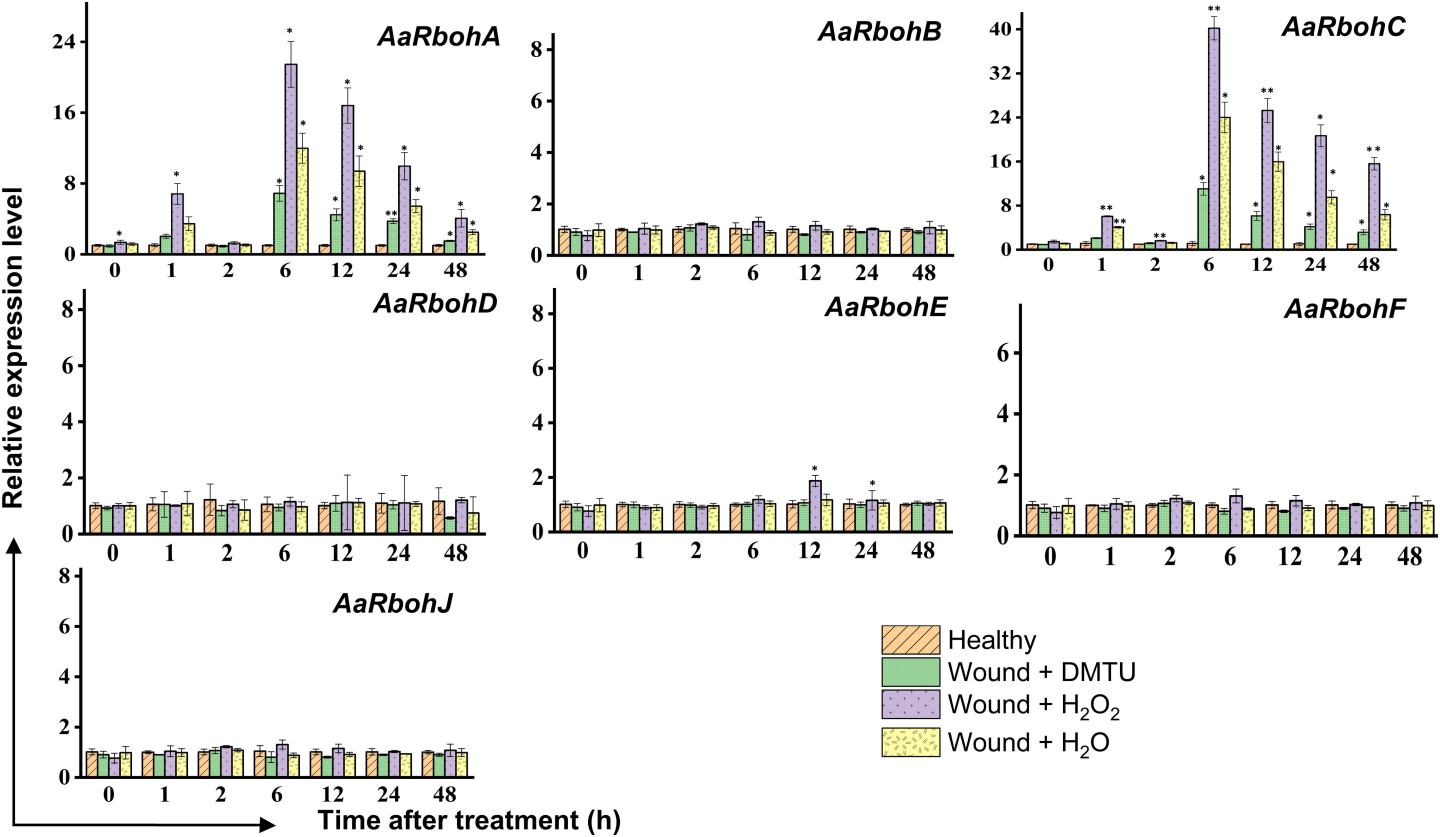
Relative expression levels of *AaRboh* genes in H_2_O_2_-treated stem of *A. agallocha*. The stems were cut, and the apical end of each cut stem was placed in distilled H_2_O, H_2_O_2_, DMTU, respectively, as appropriate. The pre-treating solution was thrown away after 2 h, and the stems were left exposed to air. The samples were taken at 0 h, 1 h, 2 h, 6 h, 12 h, 24 h, and 48 h following air exposure. The samples without any treatments are considered as healthy. Asterisks (*) denotes a significant difference compared with healthy samples at 0.05 or **P < 0.01 (Student’s t-test). Data represent means ± SE off three independent experiments.

In the wounded stem treated with H_2_O_2_, H_2_O, and DMTU, separately, the transcript levels of *AaRbohA* and *AaRbohC* experienced significant increase in H_2_O_2_-treated stem, reaching 6.82-fold and 6.05-fold, respectively, within the first hour (**Figure 10**). Subsequently, after 2 h, their expression levels returned to the initial baseline. However, at the 6-h time point, both genes exhibited a remarkable surge in expression, with *AaRbohA* and *AaRbohC* showing increase of 21.64-fold and 40.21-fold, respectively. This heightened expression subsided from 12 h and progressively declined during the 48 h of air exposure. In contrast, the treatment with water (H_2_O) resulted in a peak in the level of both genes, *AaRbohC* and *AaRbohA*, at 6 h, and their expression had not reverted to pre-treatment levels even after 48 h. However, when wounded stems were treated with DMTU, the expression of both genes decreased by about three-fold and four-fold compared to wounded stems treated with H_2_O_2_ (**Figure 10**). Meanwhile, *AaRbohB*, *AaRbohD*, *AaRbohF*, and *AaRbohJ* exhibited no significant deviations in their expression patterns compared to the control.

### 3.11 ROS determination

**Figure 11:**
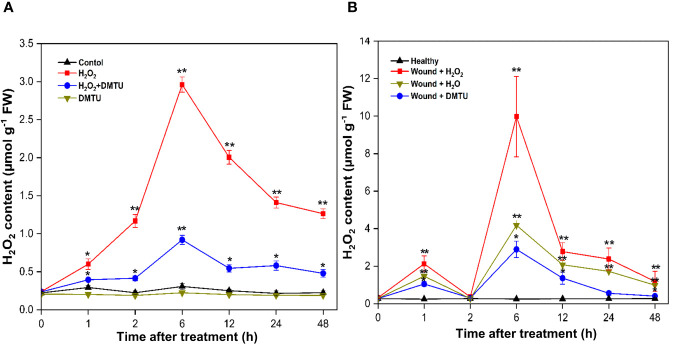
Endogenous H_2_O_2_ content in calli and stem of (*A*) *agallocha*. **(A)** Content of endogenous H_2_O_2_ in calli treated with H_2_O_2_, DMTU, and H_2_O_2_ + DMTU, respectively, for 0, 1 h, 2 h, 6 h, 12 h, 24 h, and 48 h. **(B)** Content of endogenous H_2_O_2_ in the 1-year-old stems after pruning, the cut ends were immersed in distilled H_2_O, H_2_O_2_, and DMTU. The pruned stems were exposed to air after 2 h, and the pretreating solution was discarded. The healthy condition indicates the samples without any treatment and served as control. Following air exposure, samples were collected at 0 h, 1 h, 2 h, 6 h, 12 h, 24 h, and 48 h. Asterisks (*) indicate a statistically significant difference from healthy samples at *P < 0.05 or **P < 0.01 (Student’s t-test). The data represent the means and standard deviations of three independent experiment.

The treatment with (H_2_O_2_) led to an increase in endogenous H_2_O_2_ content in both calli and actively growing wounded pruned stem tissues. In calli, a transient rise in H_2_O_2_ levels was observed at 6 h, reaching 2.96 μmol/g, after which it gradually decreased to 1.26 μmol/g by 48 h (**Figure 11A**). Moreover, treatment with DMTU alone resulted in a decrease in the accumulation of H_2_O_2_, which remained relatively constant throughout the study period. When H_2_O_2_ was applied in combination with DMTU, as expected, it led to the reduction in endogenous H_2_O_2_ production, which reached 0.92 μmol/g at 6 h. In the case of wounded stems treated with H_2_O_2_, the concentration of endogenous H_2_O_2_ experienced an initial peak at 1 h, reaching 2.12 μmol/g. Subsequently, it decreased to the baseline level at 2 h, followed by another increase. The maximum H_2_O_2_ production occurred during the second peak at 6 h, with an H_2_O_2_ concentration 31.16 times greater than the initial concentration, totalling 9.97 μmol/g. After 48 h of exposure to air, the H_2_O_2_ concentration decreased to 1.19 μmol/g. The elevated endogenous H_2_O_2_ levels were mitigated by DMTU application. Furthermore, the endogenous H_2_O_2_ content in wounded stems treated with H_2_O was lower than that in wounded stems treated with H_2_O_2_, but it was higher than that observed in the treatment with DMTU. Notably, there were no significant alteration in the endogenous H_2_O_2_ levels in healthy stems (**Figure 11B**).

### 3.12 Validation of the expression of *AaRbohA* and *AaRbohC* in naturally infected *A. agallocha* tree

**Figure 12:**
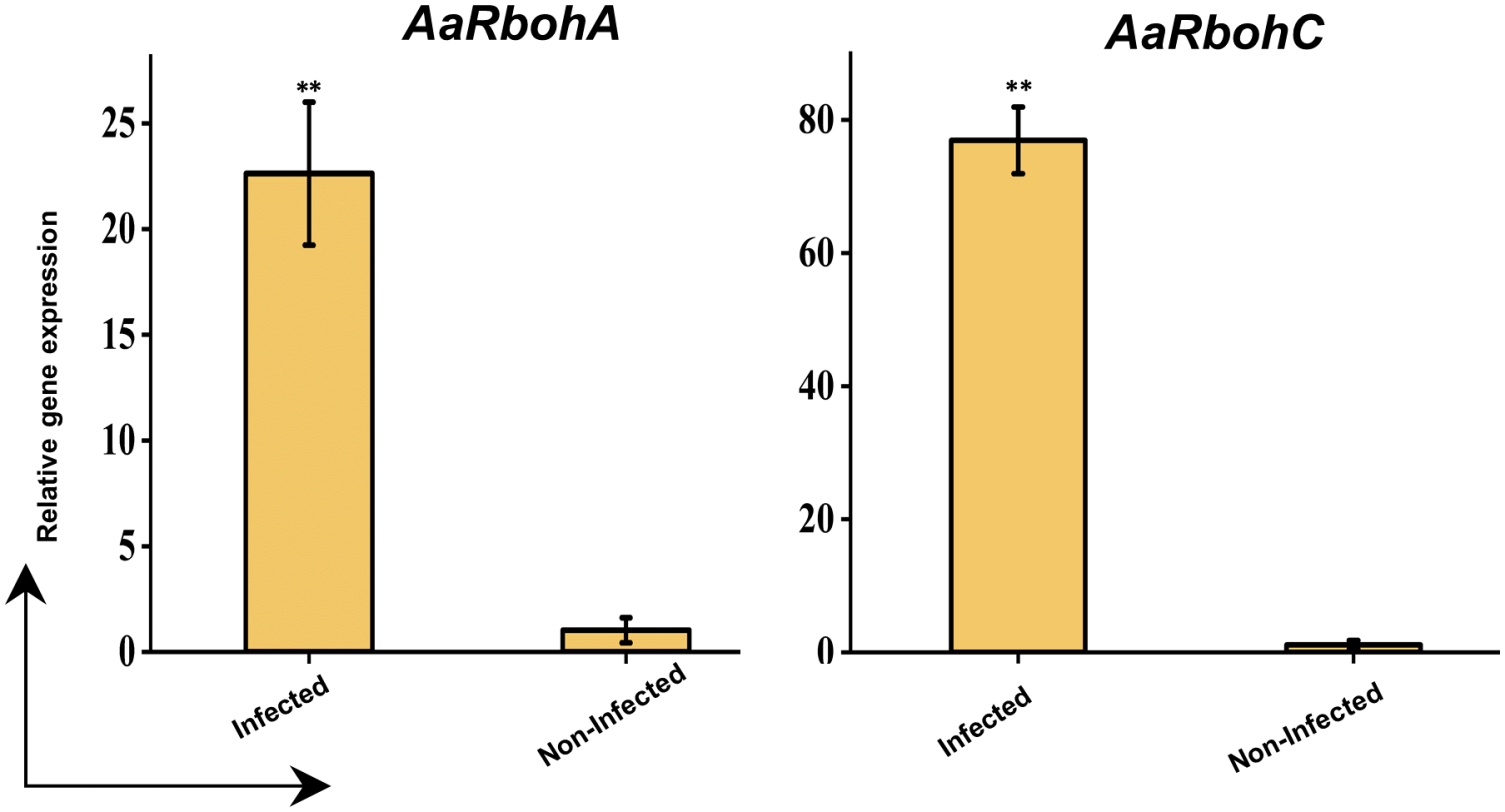
qRT-PCR analysis of two selected *AaRboh* genes. The −2^ΔΔCT^ method was used to determine relative gene expression value. The house keeping gene *GAPDH* was used to normalized the data. The * symbol indicates transcript levels that differ statistically significantly based on the student t test, and the P-value (**P < 0.01). The mean SE of three technical replicates is used to calculate each expression value. The infected and non-infected plants from Hoollongapar Gibbon Sanctuary.

The significantly higher *AaRbohC* and *AaRbohA* expression levels in both calli and stem tissues under various treatment conditions strongly suggest their involvement in stress responses. Their expression was assessed in naturally infected wood tissues to further investigate their role in response to stress. Both genes, *AaRbohC* and *AaRbohA*, exhibited substantial upregulation, with increases ranging from 22.61-folf to 76.94-fold, respectively, in the infected *A. agallocha* wood tissues compared with that in healthy wood tissues (**Figure 12**). This finding indicates the crucial role of these two members in stress responses and possibly during agarwood formation in *A. agallocha*.

## 4 Discussion

In this study, a comprehensive examination of total 14 Rboh proteins was carried out in both *Aquilaria* species. Notably, an equivalent number of 7 Rboh proteins have been reported in the genomes of several other plant species, including *Citrus sinensis* (Zhang et al., 2022), *Capsicum annuum* (Zhang et al., 2021), *Rubus occidentialis*, *Prunus dulsis* (Cheng et al., 2020), *Prunus persica* (Cheng et al., 2019), *Cucumis sativus* (Li et al., 2019), *Jatropha curcas*, *Ricinus communis* (Zhao and Zou, 2019), *Fragaria ananassa* cv. Toyonaka (Zhang et al., 2018), and *Vitis vinifera* (Cheng et al., 2013) (**Supplementary Figure 4**). This intriguing consistency in the number of Rboh proteins underscores their importance across diverse plant species. However, certain differences within the members of both the *Aquilaria* species were observed. For instance, *RbohF* and *RbohJ* were identified only in *A. agallocha*, but not in *A. sinensis*, and vice-versa in the case of *RbohH*. Similarly, maximum intron, i.e., 15 was found in *AaRbohE*, whereas 14 in *AsRbohE.* The promoter of these genes composed of various *cis*-regulatory elements, a characteristic that affirms their involvement in stress responses, hormonal regulation, and developmental processes (Jakubowicz et al., 2010; Marino et al., 2012; Huang et al., 2021; Zhang et al., 2022). Thus, aligning with previous research carried out in *O. sativa* and *A. thaliana*, the presence of these elements within the putative *Rboh* genes of *Aquilaria* indicates notable similarities in their functions (Kaur et al., 2016). In addition, plant Rboh proteins are equipped with conserved domains that facilitate their vital functions, including ROS metabolism during stress conditions, the regulation of calcium ion (Ca2+) channels, and downstream signaling processes (Torres and Dangl, 2005; Yu et al., 2020). Recent studies have delved into their roles in stress responses across various angiosperms (Hu et al., 2018; Kaur and Pati, 2018; Chang et al., 2020; Zhang et al., 2022). The results of motif analysis and homology modeling indicate that the *Aquilaria* Rboh proteins possess the essential domain NADPH_ox, in addition to a transmembrane domain likely associated with ferric reductase activity and two calcium-binding structural motifs known as EF-hand motifs specifically in RbohA and RbohC. The presence of EF-hand suggests its crucial role in interaction with small GTPases (Herve et al., 2006). These structural features strongly suggest that the ROS generated by these Rboh proteins are integral components of the cellular signaling network. The oxidative burst, characterized by the production of hydrogen peroxide (H_2_O_2_), occurs as a result of the catalytic conversion of environmental oxygen into H_2_O_2_ through the NAD(P)H oxidase H_2_O_2_ forming activity of the putative Rboh proteins (Ben Rejeb et al., 2015; Wang et al., 2018). These conserved domains in the putative Rboh proteins imply their potential involvement in stress-induced ROS generation during exposure to various stressors by *Aquilaria* species. Interestingly, few motifs in the Rboh members (except RbohA and RbohC) of *A agallocha* were missing. Overall, the presence or absence of specific motifs and differences in certain characteristics in their gene or protein sequence may linked to functional divergence and conservation of *Aquilaria* Rboh members.

Gene duplication processes significantly influence protein families’ expansion and contraction to fulfill plants’ physiological requirements (Zhang et al., 2021). Duplication events have been identified as a major force behind the expansion of *Rboh* gene family across various plant species, including *Brassica rapa* (Li et al., 2019), *Musa acuminate* (Ying et al., 2020), *Gossypium hirsutum* (Wang et al., 2020), and *A. thaliana* (Zhou et al., 2020). Our current investigation identified a single instance of segmental duplication and TRD in *A. sinensis*. However, the TRD was found to be major force of expansion of Rboh family of *A. agallocha*. The result indicated that *AaRbohA* acted as the old parent copy and generated *AaRbohB*, *AaRbohC*, and *AaRbohE* in different divergent time. The presence of intron regions in these genes also indicated their possible generation by transposon-mediated event. Similar results have been reported in case of TRAM/LAG/CRN8 (TLC) genes in maize, where Whole genome duplication (WGD) and TRD contributed to expansion and diversification of the protein families (Si et al., 2019). A significant number of genes have also been shown to undergo TRD in *A. thaliana* (Wang et al., 2013). The *Ka*/*Ks* ratio of the duplicated gene pairs in this study was found to be <1, suggesting that these duplicated genes have undergone robust purifying selection during the evolutionary course. Furthermore, the reduction in the number of Rboh proteins in both *Aquilaria* genomes, compared to *Arabidopsis* Rboh family, implies that the loss of function events possibly transpired during the evolutionary course of the *Aquilaria* genomes. Alternatively, these gene losses could be attributed to functional redundancy (Espinosa-Cantú et al., 2015; Martin and Schnarrenberger, 1997).

KEGG pathway analysis and model interaction network predictions further substantiate the role of *AaRboh* and *AsRboh* genes in plant–pathogen interactions, hormone signaling, and MAPK pathway. Rboh proteins in *H. vulgare*, *G. barbadense*, *Z. jujube*, *J. curcas*, and *M. sativa* have been shown to perform function through generation of ROS, thereby conferring protection against invading plant pathogens (Trujillo et al., 2006; Zhao and Zhou, 2019; Cheng et al., 2020). But, a study comprehending on the ROS generation and associated Rboh proteins in the genomic-level is missing in *Aquilaria* plant. Hence, within the framework of this study, the *in silico* expression analysis has elucidated the differential upregulation of *RbohA* and *RbohC* in both plant species, as well as the elevation of *RbohB* expression in *A. sinensis*. To corroborate these findings, we validated their expression levels in H_2_O_2_-mediated stress-induced callus and stem tissues of *A. agallocha* using qRT-PCR, followed by quantifying the ensuing ROS generation. An increase in the expression levels of *AaRbohA* and *AaRbohC* significantly, coupled with the maximal accumulation of ROS in both callus and stem tissues following a 6-h exposure to H_2_O_2_, provides compelling evidence of their involvement in ROS production in response to stress stimuli. In addition, the elevated expression of *AaRbohB* and *AaRbohE* exclusively in callus tissue suggests their specialized role in ROS generation, particularly within the callus. However, in *A. thaliana*, *RbohD* and *RbohF* expressed in all tissue (Orman-Ligeza et al., 2016). But in this study, we have not detected any differential expression of these members. Nevertheless, in *Solanum melongena*, *RbohB* and *RbohC* were highly expressed in leaves (Du et al., 2023). The results of the expression study corroborates previous findings about accumulation of endogenous H_2_O_2_ under salt stress conditions in *Aquilaria* itself (Wang et al., 2018). In similar lines, in *C. annum L*., cold, drought, and salt stresses have been shown to trigger significant endogenous H_2_O_2_ production via Rboh enzymes (Zhang et al., 2021). Application of stress via methyl jasmonate was able to substantially upregulate expression of *Rboh* genes in *A. sinensis* calli (Xu et al., 2013); similar observation was reported in *Nitraria tangutorum* (Yang et al., 2012). In light of these findings, it is evident that, under stress conditions, *Rboh* genes, specifically *AaRbohA* and *AaRbohC*, are upregulated and play a pivotal role in ROS generation, which may be closely linked with stress responses and hormonal regulation in *A. agallocha*.

The generation of agarwood resin that is laden with a diverse array of fragrant metabolites when *A. agallocha* is subjected to injury and biotic stress is a well-established fact (Ahmead and Kulkarni, 2017; Gao et al., 2019; Huang et al., 2022). The mechanism initiated by infection and the engagement of the MAPK signaling pathway, coupled with the orchestration of defense responses through a network of hormonal biosynthesis and modulation of terpenoid pathway genes by transcription factors, such as WRKY and MYC2, have been postulated as fundamental components intricately involved in the biosynthesis and regulation of these aromatic metabolites in agarwood (Xu et al., 2013; Lv et al., 2019; Tan et al., 2019). Interestingly, recent research on similar line suggested plant–pathogen/microbial interaction as a factor leading to ROS-mediated activation of MAPK pathway (Pacheco-Trejo et al., 2022; Chuang et al., 2022). Thus, we were interested to quantify the transcript levels of *AaRbohA* and *AaRbohC*, aiming to explore whether a similar process is at play in the natural production of agarwood within *A. agallocha* trees. Intriguingly, the significant and distinct upregulation of both these genes compared to healthy tissue strongly suggests their pivotal role in generating ROS within the wood tissue. In the course of this study, we employed an *in silico* approach, unveiling the differential upregulation of genes associated with the MAPK signaling pathway, transcription factors, and the biosynthesis of terpene backbones. The same had also been validated and is in align with our few previous studies where we obtained differential regulation of genes encoding *MAPK*, *WRKY*, *MYC2*, and terpenoid biosynthesis through qRT-PCR in naturally infected wood (Islam et al., 2020; Das et al., 2021; Islam and Banu, 2021; Das et al., 2023). We have previously observed that naturally infected Aquilaria woods exhibited higher expression levels of the genes responsible for sesquiterpene biosynthesis, which include *ADXPS*, *AHMGR*, *AFPS*, *ASS*, *DGS*, and *ADXPR* (Islam et al., 2020). In the same way, in the infected agarwood, a higher expression of signaling genes (*MK*, *WRKY*1, and *MAPK*3), jasmonate biosynthesis genes (*MYC*2 and *LOX*), and sesquiterpenes genes (*DSS*, *DGS*, *DXPS*, *FPS*, *SS4*, and *DGS1*) was observed (Islam and Banu, 2021). An *in silico* investigation shows that the molecular mechanism behind the production of numerous types of aromatic chemicals is attributed by a AaTPS gene family (Das et al., 2021). The gene family AaCYPs, involved in sesquiterpenoids and phenylpropanoids biosynthesis, and these members were shown to be enhanced in methyl jasmonate–induced callus and infected Aquilaria trees (Das et al., 2023). In addition, in *A. sinensis*, upregulation of three sesquiterpene synthases (*AsTPS10*, *AsTPS16*, and *AsTPS19*) stem tissue has been linked to sesquiterpenes accumulation in the H_2_O_2_ pruned stem (Lv et al., 2019). These findings provide compelling evidence of a cascade of events, initiated by ROS-induced activation of the MAPK pathway, subsequently culminating in the hormonal regulation of terpenoid biosynthesis through TFs like WRKY and MYC2, contributing to the agarwood resin production in *A. agallocha* tree.

Overall, the findings of this study confirm that, under stress conditions in *Aquilaria*, *Rboh* genes play a pivotal role in ROS generation, subsequently leading to the upregulation of various genes responsible for the accelerated accumulation of specifically terpenoids and other secondary metabolites as part of the defense response mechanism (Zhang et al., 2013; Xu et al., 2016; Lv et al., 2019). The generated ROS molecules are likely to serve as signaling entities, modulating the genes involved in the biosynthesis fragrant resinous agarwood.

## 5 Conclusion

In summary, this study characterized seven Rboh genes in each *Aquilaria* species, delving into their structural and functional attributes. The comprehensive analyses of phylogenetic positions, exon–intron structures, and motif patterns highlight both divergence and conservation among *Aquilaria* Rboh members. Promoter analysis strongly indicates their active involvement in stress-related pathways. The study further suggests that *Rboh* genes are functionally linked with MAPK proteins and transcription factors, including WRKY and MYC2. The two members, *viz*., AaRbohA and AaRbohC, are likely to play a role in generating ROS and may have a significant impact on the signaling pathways associated with the biosynthesis of metabolites present in resinous agarwood. Although, the full intricate molecular mechanism underlying agarwood formation is still lacking. The functional characterization of this *Rboh* gene family is expected to expedite the understanding of the initiation of agarwood deposition in *Aquilaria* plants.

## Data availability statement

The original contributions presented in the study are included in the article/**Supplementary Material**. Further inquiries can be directed to the corresponding author.

## Author contributions

KB: Conceptualization, Data curation, Investigation, Methodology, Software, Validation, Visualization, Writing – original draft. AD: Data curation, Investigation, Methodology, Software, Validation, Writing – original draft, Writing – review & editing. RA: Methodology, Software, Writing – original draft. SA: Data curation, Validation, Writing – original draft. RK: Data curation, Validation, Visualization, Writing – review & editing, Writing – original draft. SB: Conceptualization, Data curation, Investigation, Resources, Validation, Visualization, Writing – review & editing.

## Supplementary material

Supplementary Figure 1 Flowchart of the methodology obtained in the study.

Supplementary Figure 2 Domain organization of 14 *Rboh* genes of *A. agallocha* and *A. sinensis.* The characteristics domains are displayed based upon results of putative *Rboh* SMART tool search. Blue rectangular boxes represent trans-membrane regions and small pink boxes represents low complexity regions.
