# Supplementary material for: Genome-wide analysis of respiratory burst oxidase homolog (Rboh) genes in Aquilaria species and insight into ROS-mediated metabolites biosynthesis and resin deposition
Source: Front Plant Sci. 2024 Feb 9;14:1326080. doi: 10.3389/fpls.2023.1326080 (PMC10893762; doi:10.3389/fpls.2023.1326080)
Supplement: Supplementary Figure 1 — Flowchart of the methodology obtained in the study. [file DataSheet_1.zip › Supplementary Figure 4.PDF]

Supplementary Figure 4: Details of number of Rboh genes in different plants

Plant subgroup		Species Name	Number of Rboh	Family
**Algae**	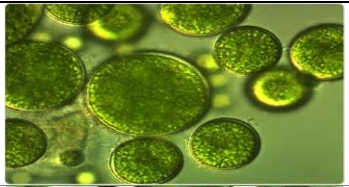	*Pyropia yezoensis*	11	Bangiophyceae
		Physcomitrelia patens	5	Funariaceae
**Bryophytes**	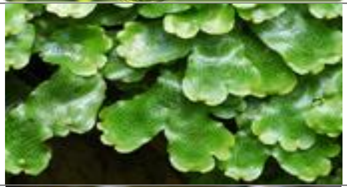	*Marchantia polymorpha*	2	Marchantiaceae
**Pteridophytes**	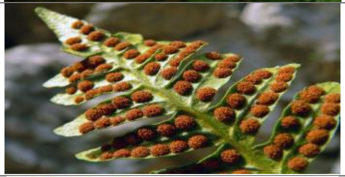	*Selaginella moellendorffii*	9	Selaginellaceae
**Gymnosperm**	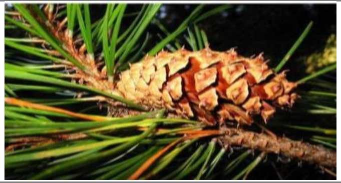	*Pinus taeda*	6	Pinaceae
		Picea abies L. Karst	13	Pinaceae
		Triticum aestivum	5	Poaceae
		Oryza sativa	9	
		Hordeum vuigare	6	
		Brachypodium distachhyon	9	
		Setaria	13	
		sorghum bicolor	10	
		Zea mays	15	
		Musa acuminara	14	Musaceae
		Vitis vinifera	7	Vitaceae

**Angiosperm**	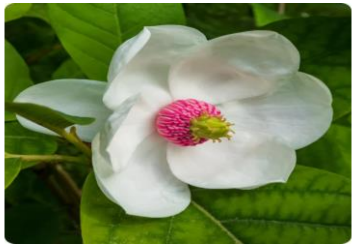	*Nymphaea colorata*	5	Nymphaeaceae
		Amborella trichopoda	5	Amborellaceae
		Vitis vinifera	7	Vitaceae
		Jatropha curcas	7	Euphorbeaceae
		Ricinus cmmunis	7	
		solanum tuberosum	8	Solanaceae
		solanum hy	7	
		Glycine max	17	Fabaceae
		Populus trichocarpa	10	Salicaceae
		Theobroma cacao	7	Malvaceae
		Fragaria vesca	7	Rosaceae
		Prunus muma	8	
		Prunus avium	4	
		Prunus persica	7	
		Prunus dulcis	7	
		Malus domestica	10	
		Brassica rapa	14	Brassicaceae
		Arabidopsis thaliana	10	
		Citrus sinensis	8	Rutaceae
		Theobroma cacao	7	Malvaceae
		Rosa chinensis	9	
