# Supplementary figures and images for: Genome-wide analysis of respiratory burst oxidase homolog (Rboh) genes in Aquilaria species and insight into ROS-mediated metabolites biosynthesis and resin deposition
Source: Front Plant Sci. 2024 Feb 9;14:1326080. doi: 10.3389/fpls.2023.1326080 (PMC10893762; doi:10.3389/fpls.2023.1326080)

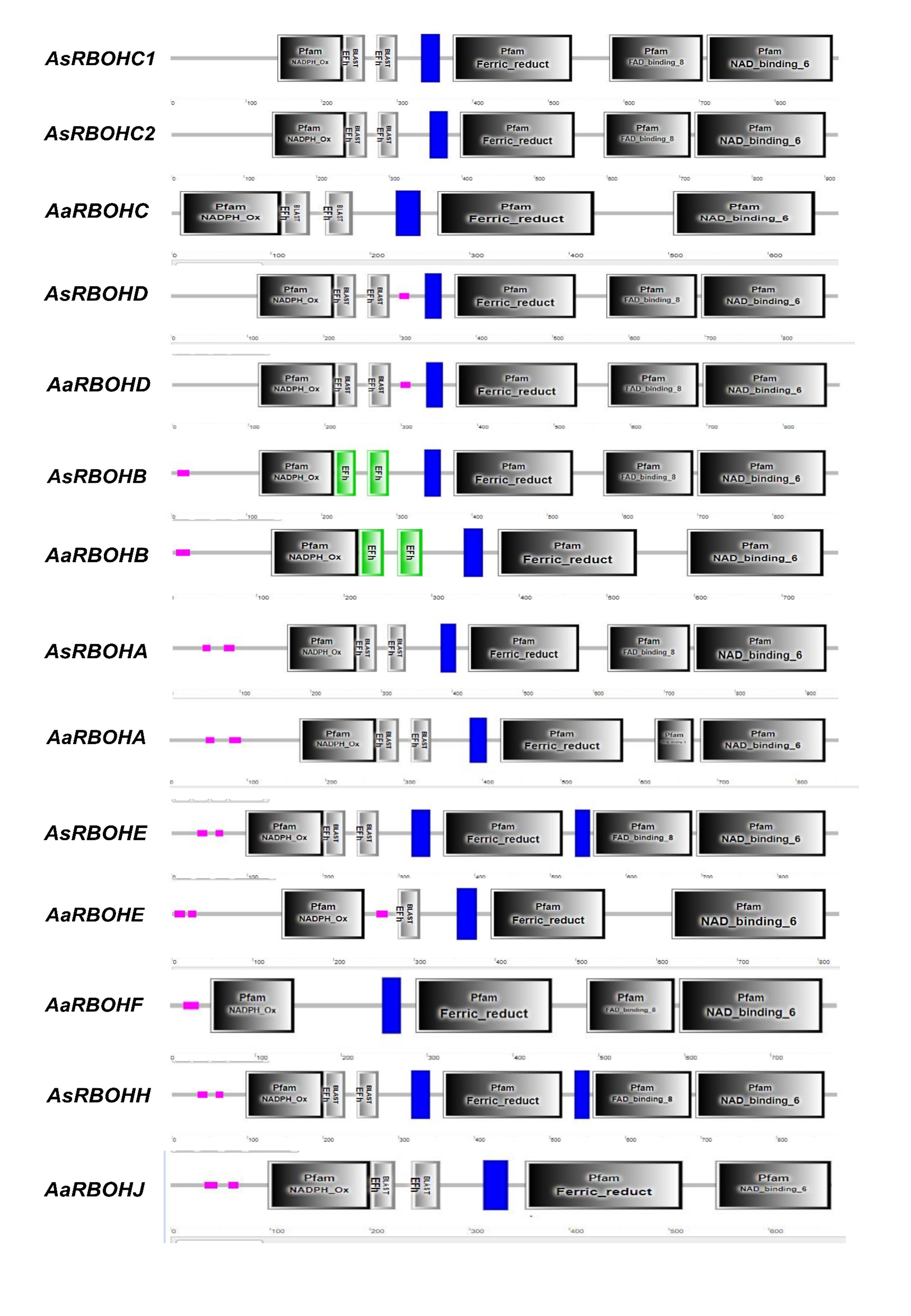

Supplement: Supplementary Figure 1 — Flowchart of the methodology obtained in the study. [file DataSheet_1.zip › Supplementary Figure 2.TIF]

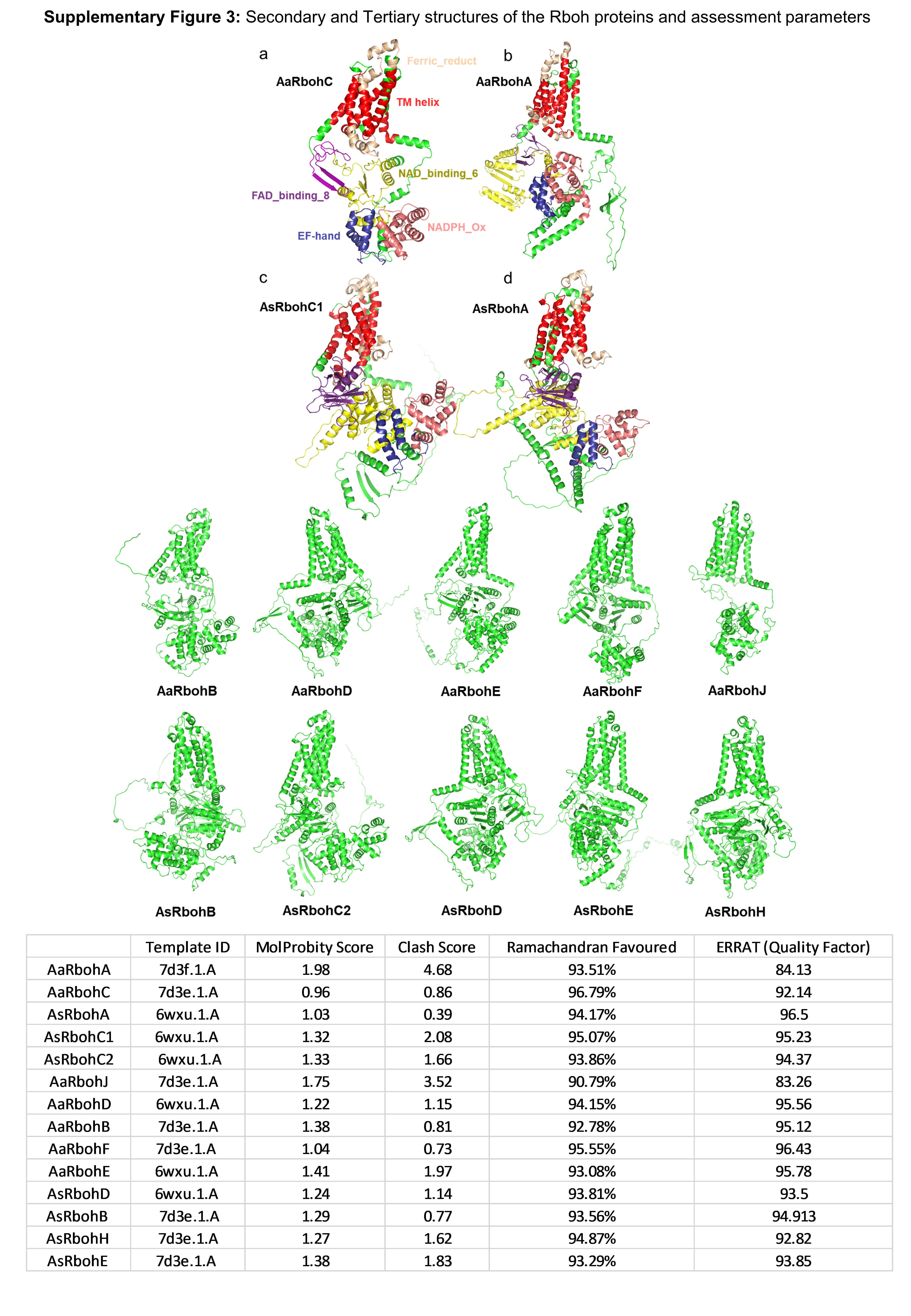

Supplement: Supplementary Figure 1 — Flowchart of the methodology obtained in the study. [file DataSheet_1.zip › Supplementary Figure 3.TIF]
